# The effect of re-warm up and its moderators on performance enhancement and rating of perceived exertion in team-sport athletes: A systematic review and three-level meta-analysis

**DOI:** 10.1371/journal.pone.0358479

**Published:** 2026-09-18

**Authors:** Jie Zhang, Honglin Tang, Xiangping Zheng, Changzhen Zuo, Yilun Xie, Yilin Fan

**Affiliations:** 1 School of Physical Education, Wuhan Sports University, Wuhan, Hubei, China; 2 Research Center for High-Quality Development of Competitive Sports, Wuhan Sports University, Wuhan, Hubei, China; Ondokuz Mayıs Üniversitesi: Ondokuz Mayis Universitesi, TÜRKIYE

## Abstract

**Objective:**

This study aimed to examine the effects of re-warm up (RWU) on athletic performance and rating of perceived exertion (RPE) in team-sport athletes and to identify potential moderators of these effects.

**Methods:**

A literature search was conducted in Web of Science, PubMed, Springer, ProQuest, SPORTDiscus, and CNKI from database inception to 7 June 2025. Three-level meta-analytic models were fitted using R software. Moderation analyses were conducted using the omnibus test method and meta-regression.

**Results:**

A total of 20 studies were included. Compared with passive rest, RWU improved countermovement jump (CMJ) performance, sprint performance, and agility, but was also associated with higher RPE (g = 1.04). No statistically significant effects were observed for squat jump (SJ) performance, repeated-sprint ability, or maximal strength. Moderator analyses indicated that takeoff style and lower-limb dominance significantly moderated the effect of RWU on CMJ performance, with significant interactions involving takeoff style, lower-limb dominance, interval format, and age group. For sprint performance, interval format significantly moderated the effect of RWU, whereas no significant moderating effects were observed for age group, running distance, BMI, RWU duration, or warm-up exercise type. The effect of RWU on RPE was significantly moderated by age group and assessment timing, with interactions involving age group, assessment timing, and interval format.

**Conclusion:**

RWU may improve CMJ performance, sprint performance, and agility, but current evidence does not indicate significant benefits for SJ performance, repeated-sprint ability, or maximal strength. Moderator effects appeared to be outcome-specific and should be interpreted cautiously, particularly where subgroup evidence was limited. Coaches are therefore advised to tailor RWU strategies to athletes’ individual characteristics, task demands, and competition schedules, while also paying attention to the risk of fatigue accumulation, particularly in youth athletes.

## 1. Introduction

Team sports, such as basketball and football, are characterised by intermittent high-intensity activities. During competition, athletes are required to repeatedly perform sprints, jumps, and changes of direction [1], all of which are essential for tactical actions such as interceptions, offensive breakthroughs, and transitions between attack and defence. Although halftime provides a necessary opportunity for recovery, passive rest during this period may lead to reductions in body temperature and neuromuscular activation [2]. This may weaken athletes’ readiness for subsequent play and contribute to performance decrements at the beginning of the second half [3–6]. To counteract this decline, re-warm up (RWU) has been proposed as an active preparation strategy during halftime [7]. Several studies have shown that RWU can improve sprint and jump performance in the early phase of the second half compared with passive rest [8–10].

In recent years, researchers have begun to synthesise the evidence on the effects of RWU. One systematic review reported that halftime RWU can help maintain physiological function and performance capacity, suggesting that it is an effective strategy for preventing performance decline after prolonged rest periods [11]. González et al. (2023) [12] conducted the only meta-analysis to date focusing on vertical-jump and sprint performance in football players and reported beneficial effects of RWU. However, the existing evidence remains limited in several respects. First, previous syntheses have focused mainly on a narrow range of performance outcomes, particularly jump and sprint performance, while key multidimensional indicators such as agility and repeated sprint ability have received less attention. Moreover, the effect of RWU on rating of perceived exertion, an important subjective outcome, has been largely overlooked. Consequently, it remains unclear whether any performance benefits of RWU are accompanied by greater perceived exertion. Second, limited attention has been paid to potential moderators of RWU effects, such as age group, body mass index (BMI), RWU duration, and interval format. This makes it difficult to determine which RWU protocols are most effective for specific athlete populations and competitive contexts. Third, existing meta-analyses have generally assumed independence among effect sizes, although studies in sports science often report multiple related outcomes from the same sample. Failure to account for this dependence may bias variance estimates and statistical inference.

To address these gaps, the present study employed a three-level meta-analysis to comprehensively examine the overall effects of RWU on multidimensional performance outcomes and rating of perceived exertion in team-sport athletes. It also explored whether athlete characteristics and RWU protocol design moderated these effects. The findings aim to provide evidence-based support for coaches and training professionals in designing more individualised and context-sensitive RWU strategies.

## 2. Materials and Methods

This systematic review and meta-analysis was conducted and reported in accordance with the Preferred Reporting Items for Systematic Reviews and Meta-Analyses (PRISMA) statement and was registered in PROSPERO (CRD420251108288).

### 2.1. Information sources and search strategy

A literature search was conducted between April 2025 and 7 June 2025. The following databases were searched: Web of Science, PubMed, Springer, ProQuest, SPORTDiscus, and CNKI. To enhance transparency and reproducibility, the complete database-specific search strategies, including field tags and final search strings, are provided in S1 File. Each database was searched from inception to 7 June 2025. No restrictions were applied regarding study design, publication date, language, age, or sex during the search process. No language restrictions were applied during the subsequent screening and eligibility assessment stages. In addition, the reference lists of relevant studies and previous meta-analyses on RWU were manually screened to identify further studies related to the research topic.

### 2.2. Selection criteria

Studies were eligible if they met the following criteria: (1) participants were adolescent or adult team-sport athletes, or completed exercise protocols designed to simulate team-sport demands; (2) the study was a randomised controlled trial, including eligible crossover designs, in which RWU was performed during half-time of a simulated or actual match; (3) the comparator involved passive rest during half-time; (4) outcomes included objective performance measures or RPE; and (5) sufficient data were available to calculate effect sizes, including means, standard deviations, and sample sizes.

Studies were excluded if they met any of the following criteria: (1) the participants were not team-sport athletes, and the exercise protocol was unrelated to team sports; (2) no passive-rest comparator was included; (3) no washout period was reported for crossover designs; (4) the study was a systematic review, meta-analysis, study protocol, grey literature, or conference abstract only; or (5) the necessary data could not be obtained.

### 2.3. Selection process

One independent reviewer (HL-T) used Zotero 7.0 to remove duplicate records from the search results. Two researchers (HL-T and J-Z) then independently screened the titles and abstracts of all retrieved studies according to the predefined inclusion and exclusion criteria. The screening focused on five key aspects: study population, intervention, comparator, outcome measures, and study design. Subsequently, the same two researchers independently reviewed the full texts of potentially eligible studies. Any disagreements were resolved through discussion, and, when necessary, a third author (XP-Z) was consulted. All disputed cases and their resolutions were documented and archived, as detailed in S11 File. After consensus was reached, the final set of included studies was determined.

### 2.4. Data extraction

Two reviewers who participated in study screening independently extracted the required data using predefined extraction criteria. Inter-rater agreement for data extraction was assessed using Cohen’s kappa coefficient [13], which showed good agreement (kappa = 0.70). This indicated good agreement between reviewers during data extraction.

When data were missing or unclear, the authors classified the missing information according to its type and importance. Studies were excluded if key information, such as study design or outcome measures, was unavailable. When non-critical information, such as training age or ambient temperature, was missing, studies were retained if the missing data did not affect calculation of the primary effect sizes; however, they were excluded from moderator analyses requiring those variables. For studies with missing data, the corresponding authors were contacted by email. If no response was received and the required data were presented only in graphical format, WebPlotDigitizer 4.7 was used to extract the data [14]. This tool has been shown to have good reliability and validity [15,16] and is widely used for data extraction in this field. Data extracted independently by two reviewers were cross-checked, with discrepancies verified by a third reviewer when required.

Extracted data included study characteristics; participant characteristics (age, sex, height, body mass, and training experience); RWU characteristics (exercise type, duration, and intensity); outcome data, including pre- and post-intervention means and standard deviations; and study design.

### 2.5. Statistical analysis

#### 2.5.1. Data synthesis.

Change-from-baseline scores were used for effect-size calculation, based on the mean change and standard deviation (SD) of the change in the RWU and control conditions. The calculation formula was as follows:


$$\begin{array}{c} {\text{Mean}}_{\text{change}}={\text{Mean}}_{\text{Post}}-{\text{Mean}}_{\text{Pre}} \end{array}$$ (1)

$$\begin{array}{c} {\text{SD}}_{\text{change}}=\sqrt{{\text{SD}}_{\text{Pre}}^{\text{2}}+{\text{SD}}_{\text{Post}}^{\text{2}}-\left(\text{2}\times \text{R}\times {\text{SD}}_{\text{Pre}}\times {\text{SD}}_{\text{Post}}\right)} \end{array}$$ (2)

According to the recommendations of the Cochrane Handbook for Systematic Reviews of Interventions [17] and Follmann et al. (1992) [18], when the pre–post intervention correlation coefficient was not reported in the original studies, r = 0.5 was used as a reasonable default value. To examine whether the assigned correlation coefficient influenced the analytical results, sensitivity analyses were conducted using three correlation coefficients: r = 0.3, 0.5, and 0.7 [19]. The results showed that the direction of the pooled effect sizes remained consistent across different correlation coefficients, and the statistical significance of the outcomes did not change, as detailed in S10 File. Accordingly, r = 0.5 was used in the primary analysis to estimate the SD of pre–post changes.

#### 2.5.2. Three-level meta-analysis.

In studies of athletic performance, multiple outcome indicators are often reported, including speed, agility, and vertical jump height. Each outcome may also be assessed using different measurement methods. For example, speed can be evaluated using 10-m, 20-m, or 30-m sprint tests, while jump performance can be assessed using countermovement jump (CMJ) or squat jump (SJ) tests. In the present study, each outcome indicator was extracted as an independent intervention comparison where applicable, resulting in multiple effect sizes from different tests and a multilevel nested data structure. Effect sizes derived from multiple outcomes within the same study are statistically dependent. Analysing such effect sizes with a model that assumes independence may underestimate uncertainty and lead to inappropriate statistical inference.

To address this issue, the present study adopted the three-level meta-analytic approach proposed by Assink and Wibbelink [20]. This method decomposes variance into sampling variance (Level 1), within-study variance (Level 2), and between-study variance (Level 3), while accounting for the correlation and hierarchical structure of effect sizes. This approach allows within-study dependence among effect sizes to be modelled explicitly and can improve estimation of uncertainty. In addition, separate datasets were constructed and analysed according to outcome type. In the three-level model, Level 2 variance represented within-study heterogeneity among effect sizes, while Level 3 variance represented between-study heterogeneity.

Three-level meta-analyses were conducted using the “metafor” package in R software (version 4.4.2). The analytical procedures were as follows. For each eligible comparison, Hedges’ g and its sampling variance were calculated. Pooled effects of RWU were estimated using the rma.mv function. The magnitude of Hedges’ g was interpreted as follows: g ≤ 0.2 indicated a trivial or weak effect, 0.2 < g ≤ 0.5 indicated a small effect, 0.5 < g ≤ 0.8 indicated a moderate effect, and g > 0.8 indicated a large effect. In the present study, k represents the number of effect sizes (i.e., intervention comparisons) included in each analysis.

To limit model complexity and reduce the risk of overfitting, multivariable moderator models included only variables that were statistically significant in univariate analyses and were considered theoretically relevant. Model parameters were estimated using restricted maximum likelihood (REML) [21], and statistical tests and 95% confidence intervals were calculated based on the t-distribution.

One-sided likelihood-ratio tests (LRTs) were used to assess whether the Level 2 and Level 3 variance components differed significantly from zero. When the P/2 value was less than 0.05, the corresponding variance component was considered statistically significant, indicating significant heterogeneity at Level 2 or Level 3 and supporting the use of the three-level model over a conventional two-level model [14]. The degree of heterogeneity was classified according to the I² value as follows: 0% indicated no heterogeneity, 25%–50% indicated low heterogeneity, 50%–75% indicated moderate heterogeneity, and >75% indicated high heterogeneity.

#### 2.5.3. Moderator analyses.

To examine moderation effects and potential sources of heterogeneity, moderator analyses were conducted based on the three-level meta-analytic model. In addition, to explore potential nonlinear relationships between continuous moderators and RWU effect sizes, restricted cubic splines (RCS) were used for nonlinear meta-regression analyses. RCS does not require a predefined functional form and can flexibly model curvilinear patterns in the data [22]. Its boundary constraints also improve the stability of estimation; therefore, RCS was adopted as an objective approach for testing nonlinear associations.

Previous studies have suggested that age, BMI, interval format, warm-up duration, intensity, and exercise type are important moderators of RWU effects. Specifically, adolescent athletes may demonstrate faster neuromuscular recruitment, whereas adult athletes may require a longer warm-up period to reach an optimal thermal state [23]. BMI may influence muscle contraction efficiency and thermoregulatory capacity, as individuals with higher BMI may dissipate heat more slowly and show different muscle metabolic responses [24]. These observations suggest that warm-up intensity and recovery duration may need to be tailored to athlete characteristics, although the optimal combination remains uncertain [21,25].

Based on the above considerations, the moderator variables in this study mainly covered two dimensions: demographic factors and training-related factors (Table 1). The demographic variables included age, body mass index (BMI), training age, and age group. The training-related variables included warm-up exercise type, exercise intensity, duration, interval format, measurement indicator, and effect category.

**Table 1:** Variable definition table.

Category	Variable	Explanation
Demography	Age	Mean age of the participants, treated as a continuous variable.
	BMI	Body mass index, calculated from height and body mass, treated as a continuous variable.
	Training age	Number of years of formal sport training, treated as a continuous variable.
	Age group	Classified as adolescents (7–18 years) or adults (>18 years), treated as a categorical variable.
Training factors	Warm-up exercise type	Classified as specific or non-specific RWU according to its relevance to the target sport or performance task, treated as a categorical variable. Specific warm-up: Warm-up activities that closely mimic the movement patterns and physiological demands of the subsequent sport task; Non-specific warm-up: General warm-up activities with limited similarity to the target sport movements.
	Intensity	Intensity of RWU, classified as low, moderate, or high, treated as a categorical variable.
	Duration	Duration of RWU, expressed in minutes per session, treated as a continuous variable.
	Interval format	Rest format between the end of RWU and the start of subsequent exercise or competition, classified according to whether a brief post-RWU interval was included, treated as a categorical variable. With interval: A recovery period incorporating multiple brief bouts of light activity; cyclic interval is considered a subcategory of this format, characterized by repeated cycles of effort and recovery; Without interval: An uninterrupted passive rest period between warm-up completion and competition onset.
	Measurement indicators	Performance or fatigue outcome used for effect size calculation, such as sprint distance, jump type, agility test, or RPE, treated as a categorical variable.
	Effect category	Classified as immediate or non-immediate effects according to the timing of outcome assessment, treated as a categorical variable. Immediate effects: Performance outcomes assessed immediately after RWU completion and before competition; Non-immediate effects: Performance outcomes assessed during competition or at delayed time points following RWU.

### 2.6. Risk of publication bias and sensitivity analysis

Publication bias was assessed using contour-enhanced funnel plots [26] and Egger's asymmetry test. The trim-and-fill method was used to evaluate and adjust for potential publication bias in the included outcomes. A p value > 0.05 was interpreted as indicating no significant publication bias. To assess the robustness of the findings, sensitivity analyses were conducted for each athletic performance outcome using the leave-one-out method based on a three-level random-effects model. When any single study was excluded, the overall pooled results did not change substantially, indicating good stability of the findings. In addition, robustness was further examined by comparing the results obtained from conventional two-level models with those from three-level models (S8 File).

### 2.7. Risk of bias and GRADE assessment

Two researchers (JZ and HL-T) independently assessed the methodological quality and risk-of-bias (ROB) of the included RCTs using the Cochrane Risk-of-Bias Assessment Tool, with ROB2 applied to crossover trials [27,28]. The assessment covered the following five domains: (1) bias arising from the randomization process; (2) bias due to deviations from intended interventions; (3) bias due to missing outcome data; (4) bias in the measurement of the outcome; and (5) bias in the selection of the reported result. Inter-rater agreement between the two reviewers was assessed using Cohen’s kappa coefficient and showed excellent agreement (kappa = 0.99). Any disagreements were resolved through discussion and, when necessary, by consulting a third experienced reviewer (XP-Z).

The certainty of evidence for the effects of RWU was assessed using the Grading of Recommendations, Assessment, Development and Evaluation (GRADE) framework [13,29]. The assessment considered five domains: study limitations, inconsistency, indirectness, imprecision, and publication bias. Because all outcomes were based on evidence from RCTs, the initial certainty rating was set as high. The certainty of evidence was then classified as high, moderate, low, or very low.

## 3. Results

### 3.1. Characteristics of included studies

**Fig 1:**
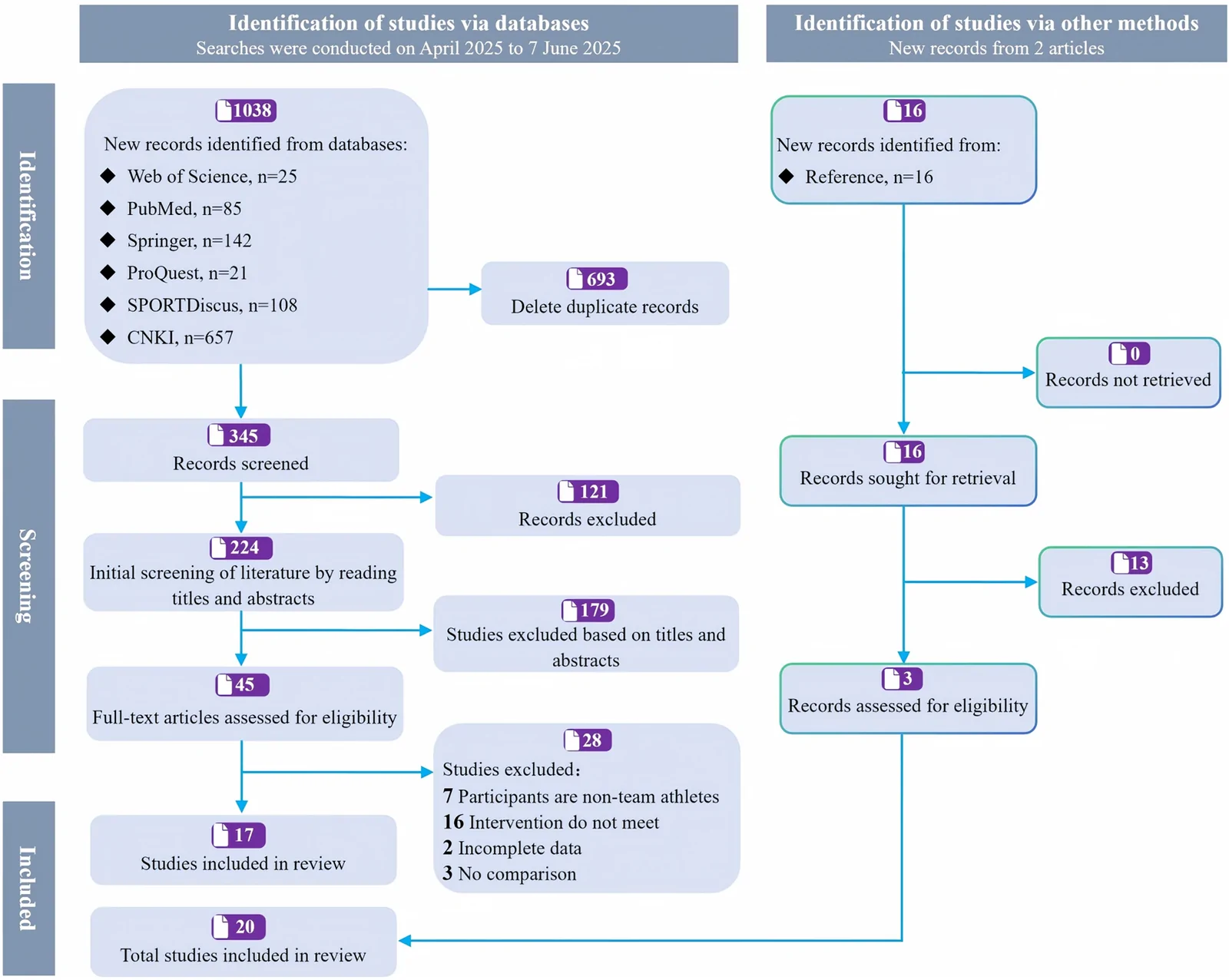
Literature screening flow chart.

After duplicate removal and title/abstract and full-text screening, 20 studies [2,7–10,30–44] were included, contributing 140 effect sizes (Fig 1). Across the included studies, there were 273 participants, with ages ranging from 13 to 25 years. The mean age was 20.42 years, and the BMI was 23.30. Regarding sport type, 52.3% of participants were football players, 18.3% were basketball players, and 3.6% were rugby players. In addition, four studies did not explicitly report the specific sports in which the participants were involved. These studies were conducted in team-sport contexts, and their activity protocols were designed to simulate the physiological or movement demands of team sports; participants from these studies accounted for 21.2% of the total sample. Characteristics of the included studies are summarised in Table 2.

**Table 2:** Characteristics of the Included Studies.

Author (year)	Participants (N)	Age (mean [SD] or range)	BMI (mean)	Sport	RWU Intensity	RWU Duration	Interval format	Outcomes	Measurement tools / units
Christaras M (2023) [30]	23	16.5 (0.4)	22.95	Football	High	3 min	Without interval	Speed; Jumps; Agility	10-m sprint; 30-m sprint; CMJ; SJ; Illinois test
Edholm P (2014) [8]	17	25 (18–33)	23.73	Football	Moderate–low	7 min	With interval	Jumps; Speed	CMJ; 10-m sprint
Koutsouridis C (2024) [31]	13	20.54 (1.45)	24.34	Basketball	Moderate–low	3 min	Without interval	Jumps; Agility; RPE	CMJ; Modified agility T-test; RPE (immediate)
Fashioni E (2020) [32]	10	23 (4)	24.34	Football	High	3 min	Without interval	Speed; Jumps; RPE	5-m, 10-m, 20-m sprint; CMJ; SJ; RPE (immediate/delayed)
Ramos-Campo DJ (2020) [33]	10	22.9 (5.3)	27.47	Rugby	Moderate–high	4.5 min	With interval	Speed; Jumps; RPE; RSA	20-m sprint; CMJ; RPE (immediate); RSA (hypoxia/normoxia)
Lovell R (2011) [9]	10	20 (1.0)	23.86	Football	Moderate	5 min	With interval	Speed; Jumps; Strength	10-m sprint; CMJ; Eccentric hamstring peak torque
Ltifi MA (2023) [34]	20	14.55 (0.51)	20.45	Football	High	3 min	With interval	Speed; RPE	20-m sprint; RPE (immediate)
Matsentides D (2023) [35]	10	18.2 (1.7)	22.02	Football	High	4 min	With interval	Jumps; Agility	CMJ; T-test agility
Mohr M (2004) [7]	16	EG: 26.07 (0.5); CG: 25.87 (1.4)	23.10	Football	Moderate	7 min	With interval	Speed	30-m sprint
Sanchez-Sanchez J (2024) [36]	20	15.7 (0.8)	23.46	Football	Moderate–low	5 min	Without interval	Vertical jumps; Horizontal jumps; Speed; Agility	SJ; SJd; SJnd; CMJ; CMJd; CMJnd; Hop jump distance; Triple hop distance; 20-m sprint; T-test
Tong TK (2019) [37]	9	20.6 (0.9)	22.72	Football / Handball	Moderate–low	4 min	Without interval	RSA; RPE; Blood lactate	RSA; RPE (immediate/in-match); [La]
Yamashita Y (2022) [38]	10	21 (2.0)	22.02	Simulated team sport	High	2 min	With interval	Blood lactate; RPE	[La]; RPE (immediate/delayed)
Yanaoka T (2018a) [10]	13	22.4 (2.1)	22.65	Simulated team sport	Moderate	7 / 3 min	With interval	RPE	RPE (immediate/delayed)
Yanaoka T (2018b) [39]	11	22.7 (2.4)	21.82	Simulated team sport	Moderate	3 min	With interval	RPE	RPE (immediate/delayed)
Yanaoka T (2021) [40]	12	22 (2.0)	22.40	Team sport player	High	1 min	With interval	RPE	RPE (immediate/delayed)
Yanaoka T (2020) [41]	12	23 (2.0)	23.43	Simulated team sport	Low / High	3 / 1 min	With interval	RPE; Speed	RPE (immediate); Sprint power
González-Devesa D (2023) [42]	10	13.7 (0.8)	20.85	Basketball	High	3 min	With interval	Jumps; RPE	CMJ; RPE (immediate)
Flórez-Gil E (2025) [43]	15	16.7 (0.6)	21.83	Basketball	Moderate–low	1 min	Without interval	Jumps; Agility	CMJ; DJ; COD speed
Yang YR (2025) [2]	12	20.5 (1.6)	24.28	Basketball	Moderate–low	4 min	With interval	Blood lactate; RPE; Agility; Jumps; Strength	RPE (immediate); T-test COD; CMJ; SJ; Maximal strength; RFD max; RFD30–250
Zois J (2012) [44]	8	23.6 (4.1)	25.23	Football	High	15 s	With interval	RSA	Peak velocity; Mean velocity

Note. For brevity, only the first author is listed for each study. Values for age and BMI are reported as mean (SD) where available. RWU, re-warm up; CMJ, countermovement jump; SJ, squat jump; RSA, repeated sprint ability; RPE, rating of perceived exertion; SJd, squat jump with the dominant leg; SJnd, squat jump with the non-dominant leg; CMJd, countermovement jump with the dominant leg; CMJnd, countermovement jump with the non-dominant leg; DJ, drop jump; COD, change-of-direction speed; RFD, rate of force development; [La], blood lactate concentration. “Simulated team-sport context” refers to exercise protocols designed to reproduce the physiological or movement demands of team sports, such as football. Detailed descriptions of RWU protocols (including specific exercises, sets, repetitions, and recovery periods) are available in S3 File.

### 3.2. Risk of Bias and Certainty of Evidence

The risk of bias of the 20 included studies was assessed using the Cochrane ROB 2 tool across five domains: bias arising from the randomization process, bias due to deviations from the intended interventions, bias due to missing outcome data, bias in measurement of the outcome, and bias in selection of the reported result. Most studies did not report the use of allocation concealment or blinding. However, blinding may be difficult to implement in exercise-intervention trials because participants and investigators can often infer the assigned condition. The randomisation domain was judged according to the RoB 2 signalling questions and information reported in each study. Overall, all 20 included studies were rated as having “some concerns” for risk of bias. Detailed domain-level risk-of-bias assessments are provided in Supporting S4 File.

The certainty of evidence for the effects of RWU was assessed using the GRADE framework across five domains: risk of bias, inconsistency, indirectness, imprecision, and publication bias. The downgrade criteria were as follows. First, for risk of bias, no downgrade was applied when more than 75% of studies were at low risk of bias. The evidence was downgraded by one level when 25%–50% of studies were at high risk of bias, or when more than 25% of studies were rated as having “some concerns” and this was considered to affect the credibility of the results [28]. In the present review, all 20 RCTs were rated as having “some concerns”; therefore, all outcomes were downgraded by one level for risk of bias. Second, inconsistency was judged using statistical heterogeneity, as indicated by I² [45]. No downgrade was applied when I² was less than 25% or when heterogeneity could be fully explained by subgroup analyses. Evidence was downgraded by one level when I² was greater than 50%, indicating moderate heterogeneity, and by two levels when I² was greater than 75%, indicating high heterogeneity. Third, indirectness was assessed according to the PICO framework. Evidence was downgraded when important differences were present in any PICO domain. Because the RWU protocols differed substantially in duration (1–15 min), intensity (low to high), and type (specific vs. non-specific), and because 52.3% of participants were football players, indirectness was considered to be present in both the population and intervention domains. Therefore, all outcomes were downgraded by one level for indirectness. Fourth, imprecision was judged using the width of the 95% confidence interval and whether the total sample size reached the optimal information size (OIS) [46]. No downgrade was applied when the total sample size met the OIS and the 95% confidence interval did not include the zone of clinical no effect or the minimal important difference (MID) threshold. Evidence was downgraded by one level when the total sample size was below the OIS or when the 95% confidence interval included the zone of clinical no effect. Fifth, publication bias was assessed using contour-enhanced funnel plots, Egger’s linear regression test, and the trim-and-fill method [47]. No downgrade was applied when the funnel plot was symmetrical, Egger’s test showed P ≥ 0.10, and the trim-and-fill method identified no missing studies. Evidence was downgraded by one level when the funnel plot was asymmetrical, Egger’s test showed P < 0.10, or the trim-and-fill method identified potentially missing studies. Detailed certainty-of-evidence assessments for each outcome are provided in S6 File.

### 3.3. Overall effect analysis

**Fig 2:**
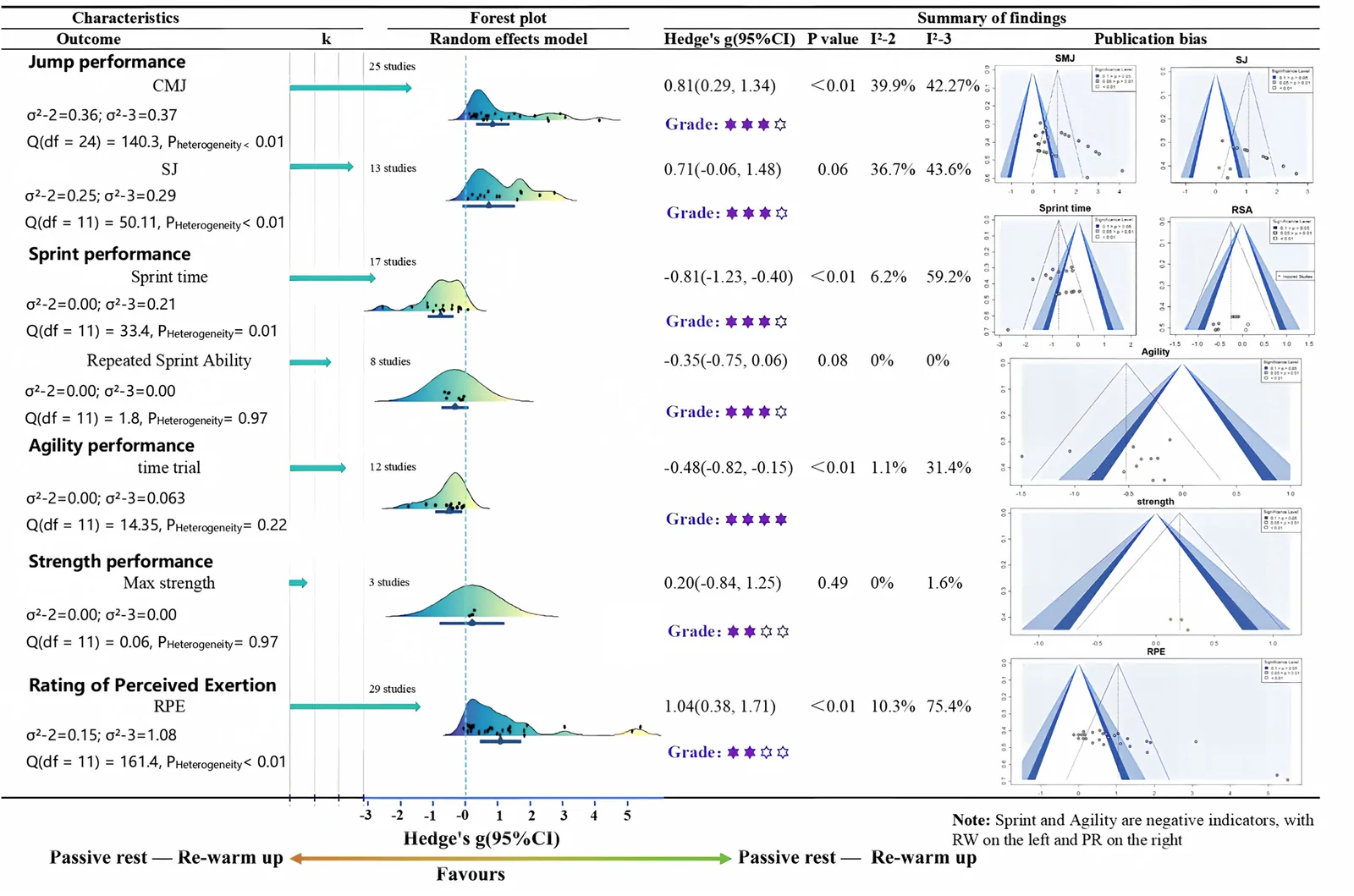
Summary of overall effects. Note: For jump performance (CMJ and SJ) and maximal strength outcomes, positive Hedges’ g values indicate superior performance in the RWU group compared with the passive rest group. For sprint and agility tests, negative Hedges’ g values indicate improved performance, as shorter completion times represent better performance. For rating of perceived exertion (RPE), positive Hedges’ g values indicate higher perceived exertion following RWU.

The overall effects of RWU on athletic performance are shown in Fig 2. For CMJ performance, RWU was associated with a significantly larger effect than passive rest (k = 25, g = 0.81, 95% CI: 0.29 to 1.34, p = 0.004), with heterogeneity distributed across Level 2 (I² = 39.9%) and Level 3 (I² = 42.27%). However, the effect on SJ height was not statistically significant (k = 13, g = 0.71, 95% CI: −0.06 to 1.48, p = 0.060). For sprint performance, RWU significantly improved sprint speed compared with passive rest (k = 17, g = −0.81, 95% CI: −1.23 to −0.40, p = 0.001), with moderate heterogeneity (I² Level 2 = 6.2%, p = 1.000; I² Level 3 = 59.2%, p = 0.020). For agility, athletes who performed RWU showed a significant improvement compared with those who underwent passive rest (k = 12, g = −0.48, 95% CI: −0.82 to −0.15, p = 0.009), with low and non-significant heterogeneity (I² Level 2 = 1.1%, p = 1.000; I² Level 3 = 31.4%, p = 0.240). RWU was associated with significantly higher RPE than passive rest (k = 29, g = 1.04, 95% CI: 0.38 to 1.71, p = 0.003), with substantial between-study heterogeneity. In contrast, RWU did not show significant effects on repeated sprint ability (k = 8, g = −0.35, 95% CI: −0.75 to 0.06, p = 0.080) or maximal strength (k = 3, g = 0.20, 95% CI: −0.84 to 1.25, p = 0.080).

### 3.4. Moderator analyses

To explore potential moderators of the overall effects of RWU and identify sources of heterogeneity across analytical levels, moderator analyses were conducted using demographic variables and training-related parameters. Only moderators that were statistically significant in univariate analyses were entered into the three-level interaction models. Given the limited number of studies, all moderator and interaction analyses should be considered exploratory. Moderator analysis was not performed for maximal strength because only three studies were included for this outcome, making the pooled effect estimate highly unstable.

#### 3.4.1. Moderators of jump performance.

**Fig 3:**
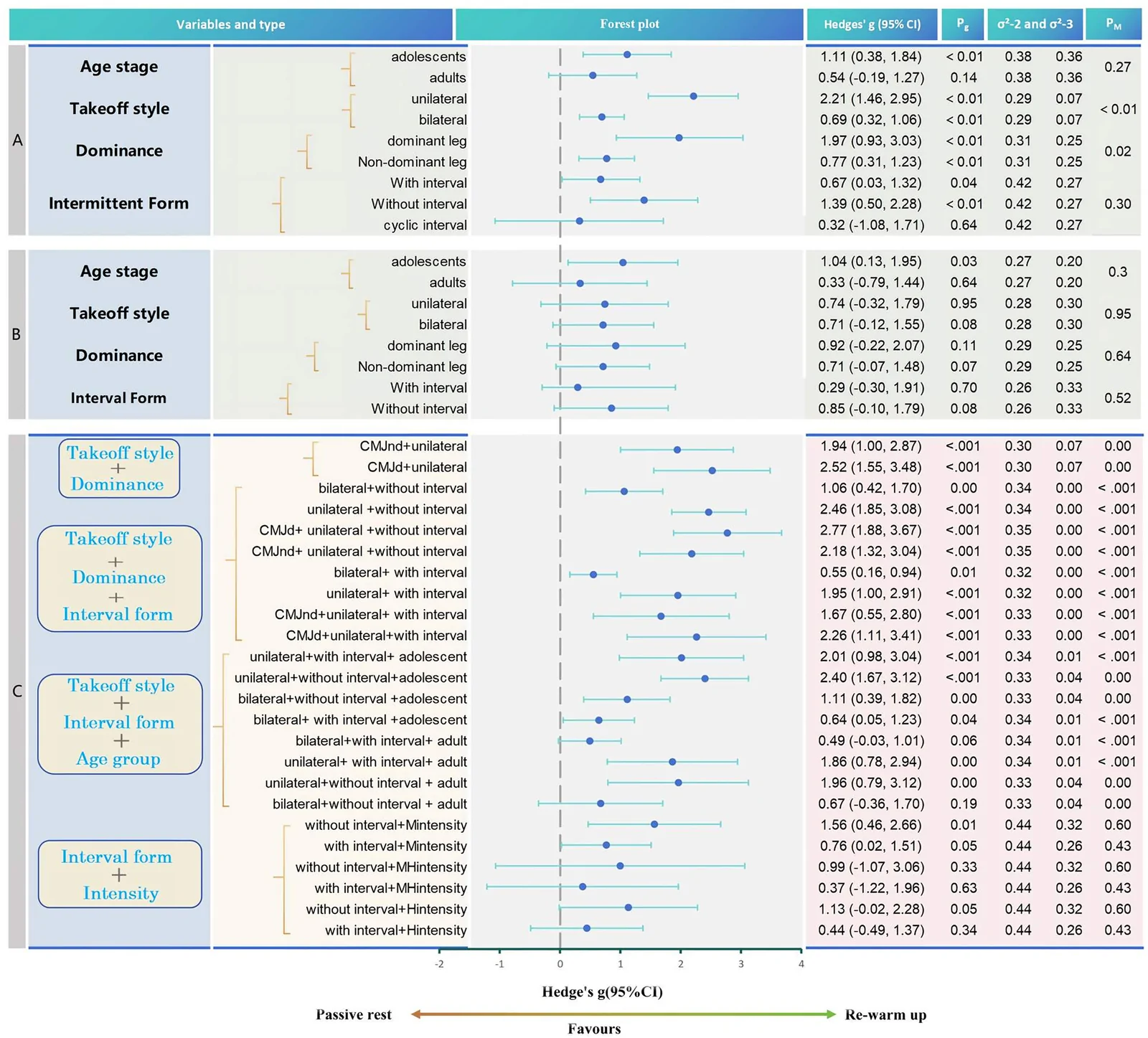
Moderating effect of re-warm up on jump performance in team-sport athletes. Note: CMJd: CMJ with dominant leg; CMJnd: CMJ with non-dominant leg; Pg indicates statistical significance compared with the control group; Pm indicates statistical significance of the moderating effect. For jump performance, positive Hedges’ g values denote enhanced performance in the RWU group relative to passive rest, whereas negative values denote reduced performance.

Moderator analysis showed that takeoff style (p < 0.01) and lower-limb dominance (p = 0.020) significantly moderated the effect of RWU on CMJ performance. Non-significant moderators included BMI, intervention duration, training age, age group, interval format, and warm-up exercise type (p > 0.05) (Fig 3; S9 File). Compared with passive rest, RWU significantly improved CMJ performance in both unilateral (g = 2.21, p < 0.01) and bilateral takeoff conditions (g = 0.69, p < 0.01), with a larger improvement observed for unilateral CMJ. Similarly, the dominant leg showed a greater improvement (g = 1.97, p < 0.01) than the non-dominant leg (g = 0.77, p < 0.01). In addition, adolescent athletes showed a significant improvement in CMJ performance after RWU during halftime compared with passive rest (g = 1.11, p < 0.01). Regarding interval format, both RWU with intervals (g = 0.67, p = 0.040) and RWU without intervals (g = 1.39, p < 0.01) were superior to passive rest during halftime. In contrast, RWU did not significantly improve CMJ performance compared with passive rest in adult athletes or under protocols with an interval (p > 0.05).

For SJ performance, although the overall effect size was not statistically significant, RWU duration emerged as a significant moderator (β = 0.63, p = 0.020). Within the 3–5 min range, each additional minute of RWU was associated with a 0.63-unit increase in the estimated effect size for SJ performance (β = 0.63; S9 File). No significant moderating effects were observed for age group, takeoff style, lower-limb dominance, interval format, BMI, training age, or warm-up exercise type (p > 0.05). In addition, adolescent athletes showed a significant improvement in SJ performance after RWU during halftime compared with passive rest (g = 1.04, p = 0.030) (Fig 3). However, no significant differences were observed among adult athletes or across subgroups defined by takeoff style, lower-limb dominance, and interval format (p > 0.05). Although warm-up exercise type was not a significant moderator, specific warm-up protocols yielded numerically larger effect estimates than non-specific protocols (S9 File).

In the sport-specific subgroup analysis, football was used as the reference category, with a pooled effect size of g = 0.951 (95% CI: 0.205 to 1.697). The pooled subgroup effect size was g = 0.730 (95% CI: −1.423 to 0.982) for basketball and g = 0.372 (95% CI: −2.516 to 1.357) for rugby. In the meta-regression model using football as the reference category, the regression coefficient for basketball relative to football was β = −0.221 (p = 0.707), whereas that for rugby relative to football was β = −0.579 (p = 0.541). Neither comparison reached statistical significance, indicating that sport type did not significantly moderate the effect of RWU on CMJ performance (S12 File).

Multivariable moderator analysis was conducted to examine interactions among variables (Fig 3; S9 File). The results showed a significant interaction between takeoff style and lower-limb dominance (p = 0.002). Specifically, during single-leg jumps, RWU produced a greater improvement in CMJ height for the dominant leg (g = 2.52, p < 0.001) than for the non-dominant leg (g = 1.94, p < 0.001).

After interval format was incorporated into the model, a significant three-way interaction was observed among takeoff style, lower-limb dominance, and interval format (p < 0.001). Specifically, across the post-RWU interval formats examined, RWU without an interval produced larger estimated CMJ effects than RWU with an interval across takeoff styles. Across all interval formats, single-leg jumps showed larger effects (interval: g = 1.95, p < 0.001; no interval: g = 2.46, p < 0.001) than double-leg jumps (interval: g = 0.55, p = 0.008; no interval: g = 1.06, p = 0.003). Furthermore, regardless of interval format, the effect of RWU during single-leg jumps was greater for the dominant leg (no interval: g = 2.77, p < 0.001; interval: g = 2.26, p < 0.001) than for the non-dominant leg (no interval: g = 2.18, p < 0.001; interval: g = 1.67, p < 0.001).

Finally, takeoff style, interval format, and age group were included in the multivariable moderator model. The results showed a significant three-way moderating effect on RWU outcomes (p < 0.01). When any two variables were controlled for, adolescent athletes consistently showed greater improvements than adult athletes, single-leg takeoff consistently showed greater effects than double-leg takeoff, and RWU without intervals consistently showed greater effects than RWU with intervals.

#### 3.4.2. Moderators of sprint performance.

**Fig 4:**
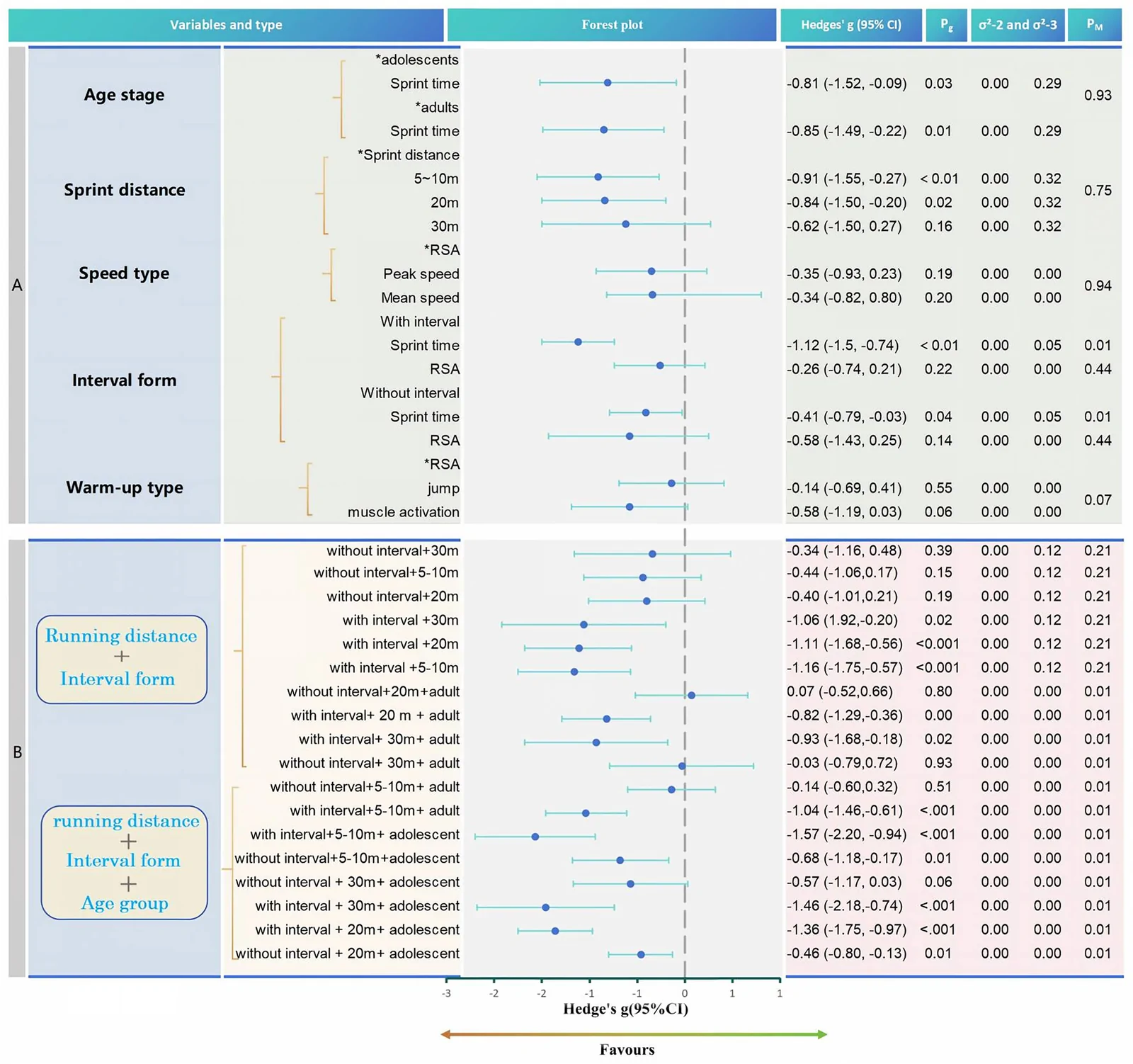
Moderating effect of re-warm up on sprint performance in team-sport athletes. Note: For sprint performance, negative Hedges’ g values denote improved performance (i.e., shorter completion time), whereas positive values denote impaired performance (i.e., longer completion time). Pg indicates statistical significance compared with the control group; Pm indicates statistical significance of the moderating effect.

Moderator analysis showed that interval format had a significant moderating effect on straight-line sprint performance across distances of 5–30 m (p = 0.010), whereas age group, running distance, BMI, duration, and warm-up exercise type showed no significant moderating effects (Fig 4, S9 File). A key finding was that both interval and non-interval RWU protocols improved straight-line sprint performance compared with passive rest. Specifically, incorporating a short rest period after RWU and before the start of the second half (g = −1.12, p < 0.01) produced a larger effect than RWU without a post-warm-up rest period (g = −0.41, p = 0.040). In addition, RWU significantly improved straight-line sprint performance compared with passive rest in both adolescent (g = −0.81, p = 0.030) and adult athletes (g = −0.85, p = 0.010).

Analyses by sprint distance showed that RWU significantly improved straight-line sprint performance over 5–10 m (g = −0.91, p < 0.01) and 20 m (g = −0.84, p = 0.020) compared with passive rest, whereas no significant difference was observed over 30 m (g = −0.62, p = 0.160). For repeated sprint ability, age group, sprint type, interval format, BMI, and duration did not significantly moderate the effect of RWU, and no subgroup showed a significant difference compared with passive rest. Although warm-up exercise type was not a significant moderator, the subgroup estimate was numerically larger for specific than for non-specific warm-up protocols (S9 File).

In the subgroup analysis of sprint performance, football was used as the reference category. The pooled effect size for the football subgroup was g = −0.345 (95% CI: −0.872 to 0.182). The pooled effect sizes for handball and rugby were g = −0.143 (95% CI: −0.669 to 1.525) and g = −0.586 (95% CI: −1.295 to 1.265), respectively. In the meta-regression model using football as the reference category, the comparison of handball with football yielded a regression coefficient of β = 0.202 (p = 0.362), whereas the comparison of rugby with football yielded a regression coefficient of β = −0.241 (p = 0.977). Neither comparison reached statistical significance, indicating that sport type did not significantly moderate the effect of RWU on sprint performance. However, these findings should be interpreted with caution because the number of effect sizes included in these subgroups was extremely limited (handball: k = 1; rugby: k = 2) (S12 File).

Multivariable moderator analysis showed no significant interaction between running distance and interval format (p = 0.210) (Fig 4; S9 File). However, compared with passive rest during halftime, RWU with a brief post-warm-up recovery interval significantly improved straight-line sprint performance over 5–10 m (g = −1.16, p < 0.001), 20 m (g = −1.11, p < 0.001), and 30 m (g = −1.06, p = 0.020).

Notably, after age group was added to the model, a significant three-way interaction was observed among running distance, interval format, and age group (p = 0.010). This finding differed from the results of the separate moderator analyses of age group and running distance. Specifically, when any two variables were controlled for, greater improvements in sprint performance were consistently observed among adolescent athletes, under RWU with intervals, and over the 5–10 m distance. Moreover, following RWU with a brief recovery interval, both adolescent athletes (5–10 m: g = −1.57, p < 0.001; 20 m: g = −1.36, p < 0.001; 30 m: g = −1.46, p < 0.001) and adult athletes (5–10 m: g = −1.04, p < 0.001; 20 m: g = −0.82, p = 0.002; 30 m: g = −0.93, p = 0.020) showed significantly greater improvements in straight-line sprint performance across all distances compared with passive rest.

In contrast, under RWU without intervals, adult athletes showed no significant differences in sprint performance at any distance compared with passive rest (p > 0.05). Adolescent athletes showed significant improvements only over 5–10 m (g = −0.67, p = 0.010) and 20 m (g = −0.46, p = 0.010), whereas no significant difference was observed over 30 m (p = 0.060).

#### 3.4.3. Moderators of agility performance.

**Fig 5:**
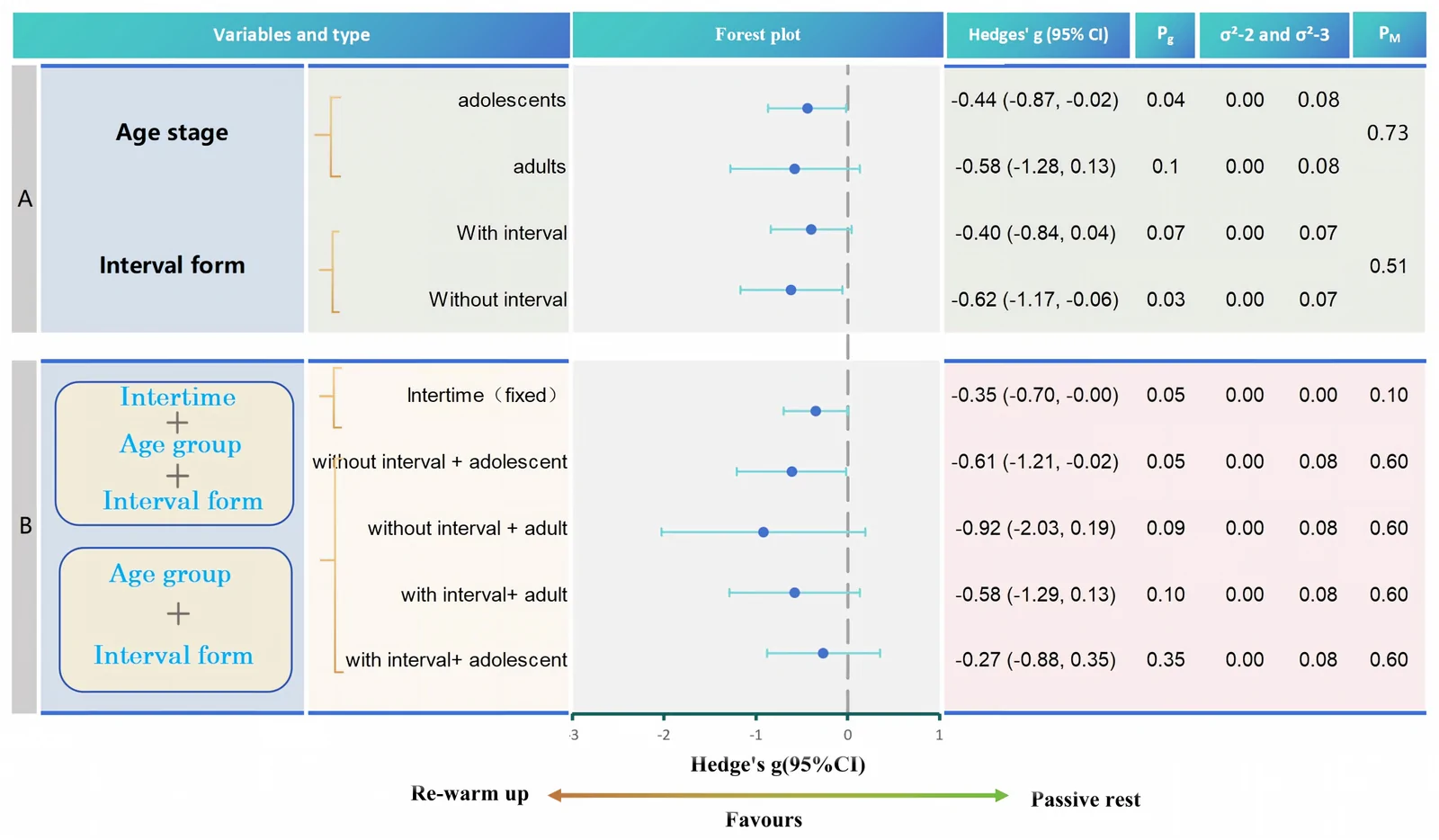
Moderating effect of re-warm up on agility performance in team-sport athletes. Note: For agility performance, negative Hedges’ g values denote improved performance (i.e., faster completion time), whereas positive values denote impaired performance (i.e., slower completion time). Pg indicates statistical significance compared with the control group; Pm indicates statistical significance of the moderating effect.

Moderator analysis showed no significant moderating effects for any of the tested variables, including age group, interval format, BMI, intervention duration, training age, and warm-up exercise type (Fig 5; S9 File). Although no moderator reached statistical significance, several descriptive subgroup patterns were observed. Adolescent athletes who performed RWU during halftime showed a significant improvement in agility performance compared with those who underwent passive rest (g = −0.44, p = 0.040), whereas no significant difference was observed among adult athletes (g = −0.58, p = 0.100). Regarding interval format, athletes who performed RWU without intervals showed significantly improved agility performance compared with passive rest during halftime (g = −0.62, p = 0.030), whereas RWU with a brief post-warm-up interval showed no significant difference (g = −0.40, p = 0.070). In addition, although warm-up exercise type did not significantly moderate agility outcomes, specific warm-up exercises showed a larger numerical effect (g = −0.51, p = 0.010) than non-specific warm-up exercises (g = −0.40, p = 0.180) (S9 File).

In the subgroup analysis of agility performance, football was used as the reference category. The pooled effect size for the football subgroup was g = −0.499 (95% CI: −1.010 to 0.011), whereas the pooled effect size for basketball was g = −0.456 (95% CI: −0.696 to 0.782). In the meta-regression model using football as the reference category, the regression coefficient for the comparison between basketball and football was β = 0.043 (p = 0.899), which did not reach statistical significance. This finding indicates that sport type did not significantly moderate the effect of RWU on agility performance. These results were consistent with the overall moderator analysis, which also identified no significant moderating effect of sport type (see S12 File).

Multivariable moderator analysis showed no significant interaction effects among the tested variables (p > 0.05) (Fig 5; S9 File). However, after controlling for age group and interval format, intervention duration showed a borderline association with the RWU effect on agility (β = −0.35, p = 0.050); given the borderline statistical evidence, this estimate should be interpreted cautiously. In addition, in the interaction analysis between interval format and age group, a significant improvement in agility performance compared with passive rest was observed only among adolescent athletes who performed RWU without intervals.

#### 3.4.4 Moderators of Rating of Perceived Exertion.

**Fig 6:**
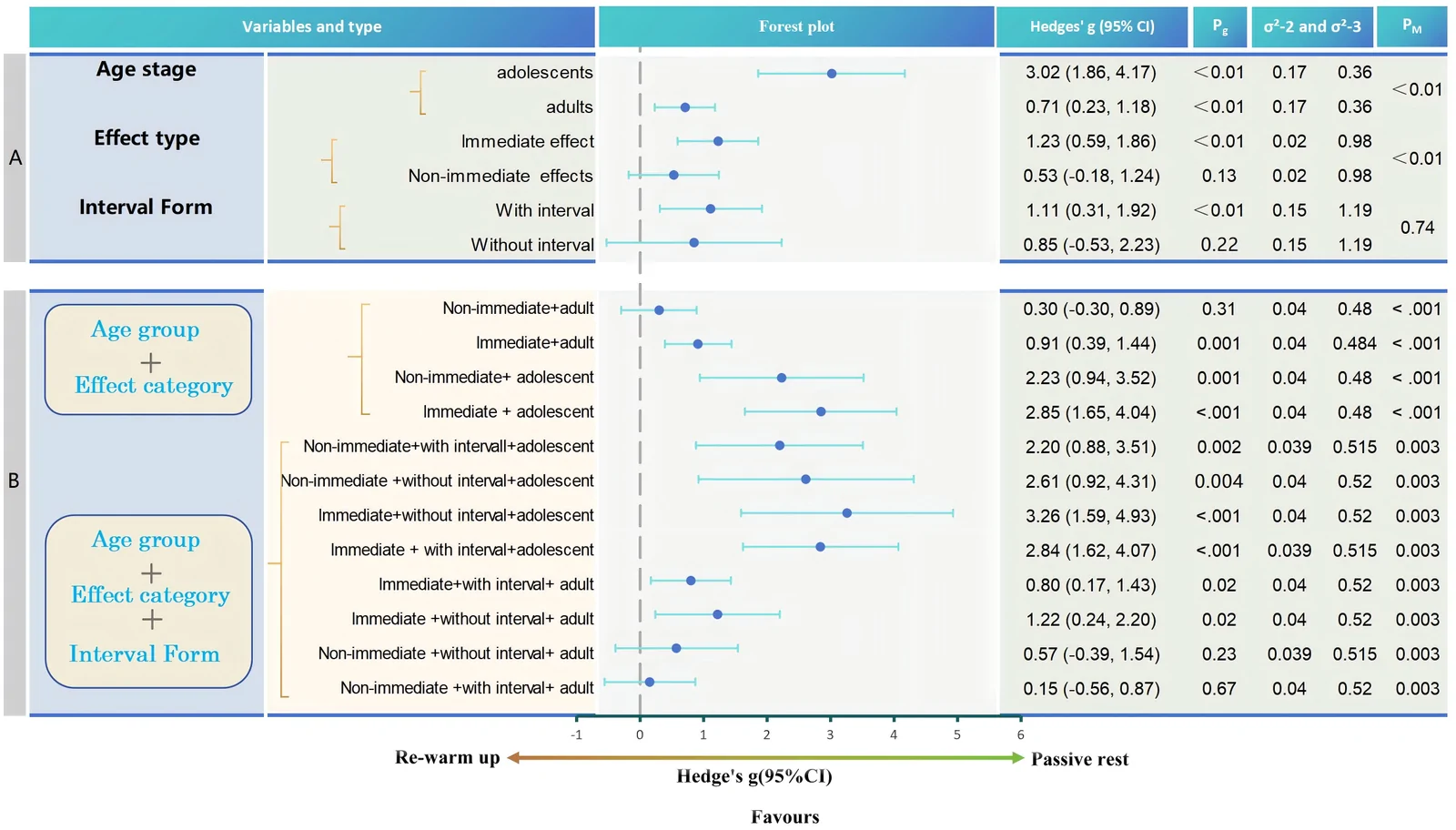
Moderating effect of re-warm up on RPE in team-sport athletes. Note: For RPE, positive Hedges’ g values denote an increase in perceived exertion following re-warm up, whereas negative values denote a decrease. Non-immediate effects: RPE assessed during the 5–45 min period of the second half; immediate effects: RPE assessed immediately after RWU; Pg indicates statistical significance compared with the control group; Pm indicates statistical significance of the moderating effect.

Moderator analysis showed that age group (p < 0.01) and effect category (p < 0.01) significantly moderated athletes’ rating of perceived exertion, whereas interval format, BMI, and duration showed no significant moderating effects (Fig 6; S9 File). Specifically, adolescent athletes showed a greater increase in rating of perceived exertion after RWU than adult athletes (adolescents: g = 3.01, p < 0.01; adults: g = 0.71, p < 0.01). In addition, across both age groups, rating of perceived exertion was higher during the immediate post-RWU period (g = 1.23, p < 0.01) than during the non-immediate period after the start of the second half (g = 0.53, p = 0.130).

In the RPE subgroup analysis, the pooled effect size for football (reference category) was g = 1.818 (95% CI: 0.244 to 3.392). The meta-regression β coefficients comparing other sports to football were: −0.974 for basketball (p = 0.386), −1.660 for rugby (p = 0.293), −1.301 for handball (p = 0.409), −0.719 for cycling (p = 0.558), and −1.012 for mixed team sports (p = 0.413). None were statistically significant, suggesting that sport type did not moderate the RWU effect on RPE. Nevertheless, findings for subgroups with only one effect size (e.g., cycling and mixed team sports) should be interpreted with caution (see S12 File).

Multivariable moderator analysis showed a significant interaction between effect category and age group (p < 0.001) (Fig 6; S9 File). Adolescent athletes showed greater increases in rating of perceived exertion than adult athletes during both the immediate post-RWU period (adolescents: g = 2.85, p < 0.001; adults: g = 0.91, p = 0.001) and the non-immediate period (adolescents: g = 2.23, p = 0.001; adults: g = 0.30, p = 0.310). This pattern suggests that younger athletes may experience a larger perceived-exertion response following half-time RWU. Importantly, adolescent athletes showed significantly higher rating of perceived exertion than passive-rest controls across all measurement periods (p < 0.01). In contrast, adult athletes showed a significant increase in rating of perceived exertion only during the immediate post-RWU period (p = 0.001), with no significant difference observed during the second half, from 5 to 45 min after restart (p = 0.310).

After interval format was added to the model containing age group and assessment timing, the overall interaction remained significant (p = 0.003). Regardless of whether a brief rest period was implemented after RWU, adolescent athletes showed significantly higher rating of perceived exertion than adult athletes and passive-rest controls during both the immediate post-RWU period and the 5–45 min period of the second half. Conversely, adult athletes showed significantly elevated rating of perceived exertion compared with passive rest only during the immediate post-RWU period, with no significant difference during the second half regardless of the rest protocol.

In the multivariable model including assessment timing, interval format, exercise intensity, and BMI, the association between BMI and the RPE effect estimate varied across intensity categories. Specifically, under high- or moderate-intensity RWU, each 1 kg/m² increase in BMI was associated with a significant reduction in rating of perceived exertion (β = −0.34, p = 0.030). Under high- or low-intensity RWU, each 1 kg/m² increase in BMI was also associated with a significant reduction in rating of perceived exertion (β = −0.37, p = 0.030). In contrast, under moderate- or low-intensity RWU, this association was no longer statistically significant (β = −0.26, p = 0.100). However, these findings should be interpreted as exploratory and hypothesis-generating. They should not be used directly to guide individualized training prescription without further confirmation from high-quality randomized controlled trials.

#### 3.4.5. Nonlinear meta-regression analysis.

**Fig 7:**
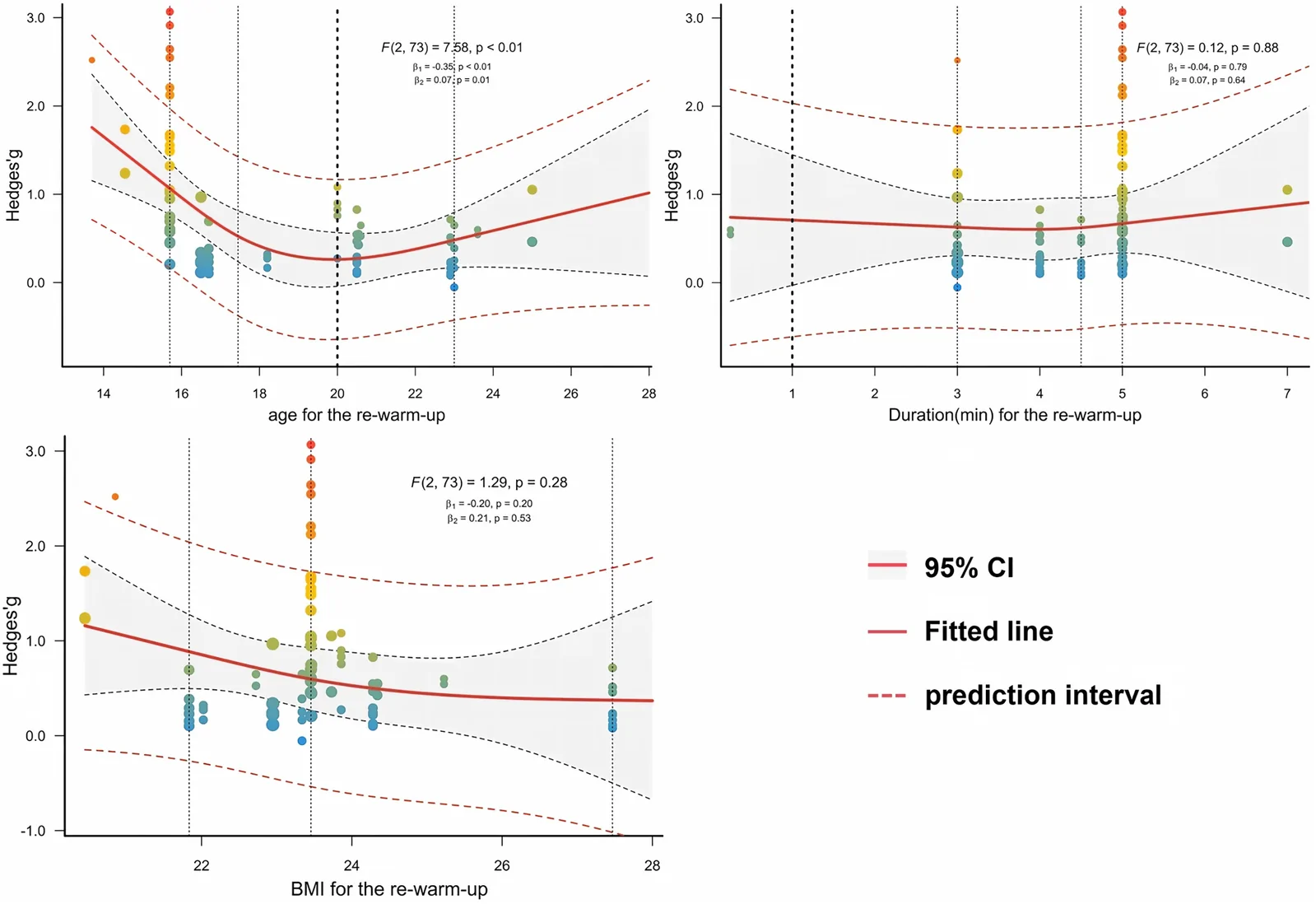
Nonlinear regression analysis of age, BMI, and warm-up duration.

To examine potential nonlinear relationships between age, intervention duration, BMI, and RWU effects, restricted cubic splines (RCS) were used for meta-regression analysis (Fig 7). The results showed a significant nonlinear relationship between age and RWU effects (F (2, 73) = 7.58, p < 0.01), characterized by a clear U-shaped curve. Specifically, among athletes younger than 20 years, age was significantly negatively associated with RWU effects. In contrast, among athletes older than 20 years, age showed a significant but weaker positive association with RWU effects, with a relatively gentle slope. No statistically significant nonlinear associations were observed for BMI (F(2, 73) = 1.29, p = 0.280) or intervention duration (F(2, 73) = 0.12, p = 0.880). The BMI and higher-order interaction findings should therefore be considered exploratory and require validation in larger, independent datasets in future studies.

### 3.5. Risk of publication bias and sensitivity analysis

Contour-enhanced funnel plots (S5 File) and Egger’s tests indicated funnel-plot asymmetry for CMJ (p = 0.010), RPE (p < 0.01), and RSA (p < 0.01), whereas no statistically detectable asymmetry was observed for SJ, sprint time, or agility. Further examination of the potential sources of bias suggested that the asymmetry observed in the CMJ and RPE funnel plots was mainly driven by outliers [36,38], which may have inflated the overall statistical significance. This indicates that the potential influence of small-study effects on the pooled estimates should be interpreted with caution. To address this issue, the trim-and-fill method was used to assess and adjust for potential publication bias. For RSA, two potentially missing studies were identified. After imputation, the effect size remained statistically non-significant (g = −0.25, p = 0.090), suggesting that these potentially unpublished studies were located in non-significant effect regions and had minimal influence on the pooled estimate. For CMJ and RPE, the trim-and-fill method did not impute additional studies; however, this does not exclude the possibility of publication bias or other small-study effects. Overall, evidence of small-study effects was outcome-specific, and publication-bias assessments should be interpreted cautiously.

Sensitivity analyses showed robust results for CMJ (g = 0.71 to 0.86), sprint time (g = −0.87 to −0.71), agility (g = −0.55 to −0.43), and RPE (g = 0.98 to 1.11). For these outcomes, all pooled effect sizes remained statistically significant after the sequential exclusion of individual studies. However, for SJ, when the indicators from the USTA group in Yang et al. (2025) were excluded, the pooled effect size changed from non-significant to significant (g = 0.81, p = 0.050), whereas the pooled effect size remained non-significant when any other study was excluded (g = 0.65 to 0.83). This sensitivity may be related to differences in task specificity, warm-up type, and participants’ fatigue status across protocols. Specifically, the USTA group in Yang et al. (2025) involved unstable-surface core training, which differed substantially from the SJ protocols used in other studies. This protocol heterogeneity may have contributed to differences in the direction or magnitude of the subgroup effect size, making the pooled SJ estimate highly sensitive to the inclusion of this group. These findings indicate that the pooled SJ estimate was sensitive to the inclusion of individual study data.

Therefore, firm conclusions regarding the effect of RWU on SJ performance should not be drawn from the current evidence.

For RSA, the small number of included studies (k = 8) and wide confidence intervals indicated limited statistical power; therefore, definitive conclusions should be avoided. Because the Level 2 variance component was estimated as zero, a two-level model was used for this outcome. The leave-one-out sensitivity analyses showed consistently non-significant pooled effect sizes (g = −0.38 to −0.30), indicating that the RSA findings were relatively stable despite the limited evidence base. Details are provided in Supporting Information 7. In addition, the comparison between the three-level model and the conventional two-level model is presented in Supporting Information 8, and the main effects and statistical significance were largely consistent between the two models. Overall, most primary estimates were relatively stable in sensitivity analyses, whereas the SJ estimate was sensitive to the exclusion of individual data.

## 4. Discussion

### 4.1. Re-warm up and athletic performance

#### 4.1.1. Jump performance.

Compared with passive rest during half-time, RWU was associated with better CMJ performance. This finding is consistent with previous studies. For example, Christaras et al. (2023) [30] reported that, after 3 min of active RWU, football players showed a significantly smaller reduction in CMJ height than those who performed passive rest. Compared with the only previous meta-analysis by González et al. (2023), the pooled effect size for CMJ observed in the present study (g = 0.81) was larger than that reported by González et al. (ES = 0.66, p = 0.001). This difference may be explained by two factors. First, the present study included a broader range of team sports, whereas González et al. included only four football studies. The larger and more diverse evidence base in the present review may have increased statistical power and captured sport-specific differences in responses to RWU. Second, the three-level model accounted explicitly for multiple effect sizes nested within studies, unlike a conventional two-level model that assumes independence among effect sizes.

Notably, active RWU did not produce a statistically significant effect on squat jump (SJ) performance. This finding differs from previous individual studies, such as Fashoni et al. (2020), which reported improvements in both SJ and CMJ after 3 min of RWU. This discrepancy may be attributable to several factors. One possible explanation is that RWU may improve performance through acute neuromuscular potentiation. Previous studies have suggested that warm-up may induce short-term neuromuscular adaptations, including increased muscle temperature, faster action potential conduction, and reduced neuromuscular signal latency [48]. These changes may enhance motor neuron excitability and muscle fibre recruitment. Because the included studies did not directly assess muscle temperature, nerve-conduction velocity, or related physiological variables, these mechanisms remain speculative. Theoretically, such changes may be more beneficial for CMJ because CMJ relies on the stretch–shortening cycle. By contrast, SJ does not involve a pre-stretch phase and may therefore be less able to utilize these potential potentiation mechanisms.

The role of muscle temperature may also be relevant. Evidence suggests that short-duration, moderate-intensity RWU may increase skeletal muscle and core temperature [7,32], thereby reducing muscle viscosity, accelerating cross-bridge cycling, increasing nerve conduction velocity, and improving explosive force production [49]. In contrast, passive rest during halftime may lead to a decline in deep muscle temperature, which is considered an important contributor to reduced jump performance [12,41]. Accordingly, RWU without an additional rest interval may help maintain muscle temperature by reducing heat loss, thereby providing a potential explanation for the observed CMJ benefits. RWU may also increase muscle temperature, heart rate, and oxygen uptake, promote muscle oxygenation, and support phosphocreatine resynthesis [7,50], providing short-term metabolic support for explosive jumping. Passive rest lacks this metabolic activation process. Nevertheless, these mechanisms remain inferential in the present study. In addition, the effect of RWU on SJ approached statistical significance (p = 0.060), suggesting that SJ may still benefit from RWU under specific protocol or participant conditions. Larger trials are needed to verify this possibility, and firm conclusions should not be drawn from the current evidence.

The moderator analyses further indicated that the effects of RWU on jump performance were condition-dependent. However, the number of studies varied substantially across subgroups, and some subgroup comparisons, such as BMI, training age, and warm-up exercise type, may have had insufficient statistical power. The confidence intervals for several interaction estimates were also relatively wide. Therefore, the following subgroup findings should be interpreted as exploratory and with caution. Univariate moderator analysis showed that takeoff style and lower-limb dominance significantly moderated improvements in CMJ height, whereas BMI, training age, interval format, and warm-up exercise type did not show significant moderating effects. The improvement in CMJ performance was greater for single-leg than for double-leg takeoff, particularly in the dominant limb. This may be related to the specific demands of team sports such as basketball and football. In these sports, single-leg actions, especially those involving the dominant leg, are used more frequently than bilateral actions. As a result, greater fatigue may accumulate in the muscles involved in single-leg actions during the first half. RWU may therefore have greater acute effects on movement patterns that are frequently used in team sports, although this mechanism was not directly tested.

Multivariable moderator analysis showed a significant interaction among takeoff style, lower-limb dominance, and interval format. Regardless of whether an interval was used, single-leg jumps consistently showed greater improvement than double-leg jumps, and the dominant leg consistently showed greater improvement than the non-dominant leg. In addition, regardless of takeoff style, RWU without a brief rest interval produced greater improvements in CMJ performance than RWU with a brief rest interval. However, because warm-up intensity was not uniformly controlled across studies, it cannot be concluded that the presence or absence of a rest interval was the sole cause of this difference. Indeed, warm-up intensity is likely to be an important factor in determining whether a short rest period is needed after RWU. No significant intensity-by-interval interaction was detected (S9 File); therefore, the available data do not establish that the relative effect of interval format differs across warm-up intensities.

This finding may be related to the maintenance of muscle temperature after RWU. Previous studies have shown that a 1°C decrease in muscle temperature may reduce athletic performance by approximately 3% [51]. RWU protocols without an additional post-warm-up rest period may help maintain muscle temperature by reducing heat loss, which may be particularly important for enhancing the contractile performance of type II muscle fibres, which play a dominant role in explosive force production [52]. Previous research has also suggested that age may moderate the benefits of warm-up in football players [53], and the present study provides further evidence for this association. We found a significant interaction among takeoff style, interval format, and age group, with RWU showing consistently greater improvements in CMJ performance among adolescent athletes under different conditions. However, because the included studies lacked direct age-group comparison designs, the age-related differences observed here should be interpreted as statistical patterns rather than confirmed causal effects. Future experimental studies are needed to clarify the mechanisms underlying these age-specific responses.

#### 4.1.2. Sprint performance.

RWU significantly improved overall straight-line sprint performance across studies using 5–30 m sprint tests. This finding differs from the recent meta-analysis by González et al. (2023) [12], which reported no significant improvement in straight-line sprint performance following RWU (g = 0.19, p = 0.440), whereas the present study found a significant overall benefit for sprint performance (g = −0.81, p < 0.01). This discrepancy may be explained by two factors. First, the previous meta-analysis included only football players, whereas the present study included athletes from football, basketball, rugby, and other team sports. Differences in running patterns, fatigue profiles, and sport-specific halftime demands may influence athletes’ responses to RWU. Second, the discrepancy may also be related to the way sprint distance was analyzed. Distance-specific analyses showed significant effects at 5–10 m and 20 m, whereas the 30 m subgroup did not reach statistical significance. In contrast, previous work did not conduct this more detailed distance-specific analysis, which may have obscured positive subgroup effects. A recent systematic review on RWU also supports the present findings and suggests that RWU can enhance sprint performance and muscle strength after halftime [11].

The potential mechanisms underlying these effects may be related to changes in muscle temperature and post-activation performance enhancement (PAPE). RWU may increase muscle temperature, thereby improving nerve conduction, muscle contraction velocity, and the time required to reach peak tension [7]. Another possible mechanism is PAPE, which refers to the acute enhancement of performance following maximal or near-maximal voluntary contractions. However, these mechanisms should be interpreted cautiously, because the included studies did not directly assess physiological indicators such as muscle temperature, phosphocreatine resynthesis, or neuromuscular activation.

In contrast to straight-line sprint performance, repeated sprint ability (RSA) showed only a small improvement and did not reach statistical significance. When the data were analyzed using a conventional two-level random-effects model, the effect appeared statistically significant. However, this result should be interpreted with caution, because the conventional model does not account for correlations among effect sizes extracted from the same study and may therefore overestimate the statistical significance of the RSA effect. For this reason, the present study used the three-level random-effects model as the basis for its conclusions. Previous studies have suggested that RSA is limited by finite energy supply and the accumulation of metabolic by-products [54]. Athletes with better aerobic fitness and faster phosphocreatine (PCr) resynthesis rates are generally better able to maintain RSA [55]. However, evidence remains mixed. Some studies have reported that active warm-up during halftime may reduce aerobic capacity compared with passive rest [11], which may partly explain the non-significant RSA result observed in the present study. Conversely, Yanaoka et al. (2018) [10] reported that RWU increased oxygen uptake (VO_2_), carbon dioxide output (VCO_2_), and oxygenated haemoglobin (Δoxy-Hb) within 10 min, suggesting activation of aerobic metabolism. This mixed evidence reflects ongoing debate regarding the relationship between aerobic capacity and RSA performance [56]. Overall, sprint responses to RWU may be influenced by multiple contextual factors, including athletes’ initial fatigue status, training level, muscle fibre composition, and pre-match warm-up protocols. Because the present study could not control for these factors or extract relevant data from all original studies, the interpretation of the RSA findings should be limited to the available evidence. The specific effect of RWU on RSA therefore requires further validation.

The present study also found that interval format significantly moderated sprint time, whereas age group, running distance, BMI, intervention duration, and warm-up exercise type did not show significant moderating effects. In contrast to the findings for CMJ, RWU protocols that included a brief rest period produced greater improvements in 5–30 m sprint performance than RWU protocols without rest. This may be related to the theoretical mechanism of PAPE. According to PAPE theory, acute performance enhancement after maximal or near-maximal voluntary contractions depends on the balance between fatigue and potentiation [7]. Nevertheless, the application of PAPE in real sport settings remains debated, and individual responses can vary substantially. Introducing a brief rest period after RWU may help reduce fatigue while allowing potentiation-related benefits to emerge. For sprint performance, appropriately timed recovery may facilitate PCr resynthesis and the clearance of metabolic fatigue. A previous meta-analysis suggested that 7–10 min of rest after moderate-intensity conditioning activities may maximize subsequent performance enhancement [55].

The multivariable analysis showed that, although the interaction between running distance and interval format was not significant, a brief rest period after RWU still significantly improved 5–10 m, 20 m, and 30 m straight-line sprint performance compared with passive rest. When age group was further included in the model, a significant interaction emerged among running distance, interval format, and age group. In the fitted interaction model, RWU with a brief interval generally yielded larger sprint effect estimates than RWU without an interval and showed significant benefits versus passive rest across several age–distance combinations. This advantage was more pronounced in adolescent athletes. These findings partly support the view that an optimal recovery period after conditioning activity may be necessary to reduce fatigue and induce PAP/PAPE-related benefits [57,58]. However, PAP/PAPE responses are highly individualized, and in complex team sport contexts, multiple physiological and psychological factors may simultaneously influence sprint performance. Therefore, PAP/PAPE theory alone may not fully explain the observed findings.

From a practical perspective, the choice between RWU with or without a brief interval should not be viewed as a simple binary decision, but rather as a matter of prioritising protocol options according to context. Although RWU with a brief interval showed the strongest overall benefit for sprint performance, RWU without an interval still significantly improved 5–10 m and 20 m sprint performance in adolescent athletes compared with passive rest. These findings highlight the strategic flexibility available to coaches when designing halftime RWU protocols. Decisions should integrate competition schedules, athlete age, recovery time, and event-specific demands in order to optimise RWU strategies.

#### 4.1.3. Agility performance.

Consistent with most previous studies, the present study showed that RWU significantly improved athletes’ agility performance compared with passive rest during halftime. This improvement may be attributable to increases in skeletal muscle temperature, enhanced central nervous system recruitment, and improved stretch–shortening cycle function, which may collectively facilitate rapid changes of direction, turning, and acceleration [8,59]. However, because the included studies did not directly measure these physiological mechanisms, these explanations should be interpreted as theoretical rather than confirmatory.

We further explored potential moderators of agility performance. Overall, age group, BMI, training age, warm-up exercise type, interval format, and intervention duration did not show significant moderating effects. Notably, subgroup analyses showed statistically significant improvements in agility performance among adolescent athletes and under RWU protocols without intervals. This may be partly due to insufficient statistical power resulting from the small number of studies in some subgroups, which may explain why significant effects were not observed among adult athletes or under RWU protocols with intervals.

After adjustment for age group and interval format, RWU duration showed a borderline association with the agility effect estimate within the observed 3–5 min range (β = −0.35, p = 0.050). Any duration-related pattern should be interpreted cautiously because the observed range was narrow and the statistical evidence was borderline. Existing evidence suggests that muscle temperature rises rapidly within 3–5 min after warm-up and reaches a relatively stable state after 10–20 min [60], providing partial support for this interpretation.

The present findings differ somewhat from those of Bishop (2003) [61], who suggested that, under moderate- to high-intensity warm-up conditions, short-term performance may decline rapidly as warm-up duration increases if no brief recovery interval is provided. Bishop also suggested that, when warm-up duration exceeds 3 min, further extending warm-up activities may have limited additional effects on subsequent performance. The discrepancy between Bishop’s conclusions and the present findings may be related to differences in warm-up intensity, participant characteristics, and the control of potential confounding factors across studies. Therefore, although the current findings support the beneficial role of RWU in improving agility performance, they should be interpreted with caution.

Overall, RWU appears beneficial for agility performance, whereas evidence that age or interval format modifies this effect remains inconclusive. Future intervention studies should further refine RWU protocols by considering exercise intensity, exercise type, age group, and interval format.

#### 4.1.4. Rating of perceived exertion.

Consistent with previous research, the present study showed that RWU significantly increased athletes’ rating of perceived exertion. Traditionally, concerns about fatigue in the second half have been one reason why coaches may be cautious in applying RWU strategies [11]. The magnitude of the RPE effect varied by age group and assessment timing. Specifically, adolescent athletes showed significantly higher rating of perceived exertion than passive-rest controls both immediately after RWU and during the later period of the second half. In contrast, adult athletes showed a significant increase in rating of perceived exertion only immediately after RWU, with no statistically significant difference during the delayed testing period. Moreover, a brief rest period after RWU did not eliminate the increase in rating of perceived exertion among adolescent athletes.

An important finding of this study is that RWU increased rating of perceived exertion while simultaneously improving several performance outcomes, including jump, sprint, and agility performance. This coexistence of increased rating of perceived exertion and performance benefit represents a central practical trade-off in halftime RWU implementation. One possible explanation is that RPE reflects perceived effort and may not closely track acute changes in physical performance capacity. At the same time, physiological responses to RWU, such as increased neuromuscular activation and muscle temperature, may support performance despite higher perceived exertion. Therefore, when designing RWU protocols, coaches need to balance the risk of immediate fatigue against potential second-half performance benefits. This is particularly important for adolescent athletes, whose fatigue responses should be monitored carefully.

Compared with previous studies, the present study also found that rating of perceived exertion may be associated with athletes’ BMI, although this finding should be interpreted with caution. In the multivariable model, the association between BMI and the RPE effect estimate was negative under selected intensity contrasts; however, these interaction estimates should be interpreted as exploratory.

Importantly, this finding does not mean that athletes with higher BMI experienced lower absolute RPE at the same exercise intensity. Rather, it suggests that the slope of RPE change with increasing exercise intensity may be flatter in athletes with higher BMI. In other words, as exercise load increased, the rate of increase in rating of perceived exertion appeared to be lower among athletes with higher BMI.

This result appears to differ from the traditional assumption that higher BMI is associated with greater exercise burden and higher rating of perceived exertion. It also differs from some previous studies that found no significant association between BMI and RPE [62,63]. However, the present finding concerns the change in RPE relative to increasing load, rather than the absolute RPE value under the same workload. In this sense, it is partly consistent with She et al. (2015) [64], who reported a negative association between BMI and RPE-related parameters. A possible mechanism may involve the generation of perceived exertion. Previous evidence suggests that RPE mainly reflects the central nervous system’s perception of motor command rather than relying solely on peripheral afferent signals, such as muscle metabolites or cardiorespiratory feedback [65]. Marcora (2009) argued that RPE may represent a conscious perception of central motor command [60]. From this perspective, RWU may increase subjective sensory load while simultaneously enhancing neuromuscular activation and muscle temperature, thereby maintaining or improving actual performance [60]. This may help explain why RPE increased after RWU while muscular performance did not decline in parallel.

Objective performance evidence also supports the possibility of a dissociation between rating of perceived exertion and performance. For example, Ltifi et al. reported that a 3 min loaded RWU significantly improved halftime sprint performance, while RPE increased only slightly [34]. This example illustrates that higher perceived exertion after RWU does not necessarily coincide with poorer acute performance. Whether the dissociation between RPE and performance differs according to BMI remains uncertain. Nevertheless, the generalisability of this interpretation across BMI groups remains uncertain and should be verified using objective indicators, such as CMJ height, sprint time, blood lactate, and other physiological or performance measures.

From a practical perspective, coaches should use objective performance indicators as the primary basis for decision-making, while using RPE as a complementary monitoring tool. When RPE increases but performance also improves, this may represent an acceptable or even desirable acute training response. If markedly elevated RPE is accompanied by deteriorating performance, coaches may consider reducing RWU intensity or allowing additional recovery. Future studies should further examine the quantitative relationship between the slope of RPE change and the rate of decline in actual performance capacity in athletes with different BMI profiles, in order to develop more individualised monitoring thresholds.

It should also be noted that the high heterogeneity observed for rating of perceived exertion (I^2^ Level 3 = 75.4%) may be attributable to differences in age group (adults vs. adolescents), effect type (immediate vs. delayed), as well as their interaction. Nevertheless, the pooled effect size and its direction remained stable across sensitivity analyses (S7 File), suggesting that the high heterogeneity did not materially affect the overall reliability of the findings. Although sensitivity analyses indicated a stable direction of effect, the high heterogeneity limits confidence in the precise magnitude and generalisability of the pooled estimate.

### 4.2. Practical significance

This study is the first to use a three-level meta-analytic model to systematically examine the effects of RWU on athletic performance and rating of perceived exertion in team-sport athletes, while also exploring potential moderating factors. The findings provide preliminary evidence-based support and practical guidance for coaches and practitioners seeking to optimise RWU strategies. However, these recommendations are based largely on exploratory analyses, and most conclusions require further confirmation through high-quality primary studies. Therefore, coaches should adapt these findings to the specific characteristics of their teams rather than treating them as universally applicable protocols.

For jump performance, coaches may consider incorporating a small number of high-intensity plyometric activities during halftime RWU and minimising the rest interval after RWU where appropriate. However, the generalisability of this approach across different sports and competitive levels remains to be tested. In sports involving frequent unilateral force production, sport-specific unilateral exercises may be considered, although the apparent dominance-related differences remain exploratory.

For sprint performance, the present findings suggest that a brief rest period after RWU may be beneficial for supporting energy-system recovery and improving subsequent sprint performance. For agility-related tasks, the available data suggest that short RWU protocols may be feasible, although the optimal duration remains uncertain. A brief, moderate-intensity RWU may offer a practical balance between activation and perceived exertion, but the optimal duration and recovery format remain uncertain. Nevertheless, the optimal RWU duration may vary according to sport type, age group, and physical fitness level. Coaches are therefore encouraged to conduct small-scale trials within their own teams before applying a protocol more broadly.

Although adolescent athletes showed larger performance and RPE responses in some analyses, the available evidence is insufficient to prescribe a specific recovery interval on the basis of age alone. If competition schedules are tight and no additional rest interval can be arranged, RWU may still be useful for adolescent athletes, particularly for 5–20 m sprint performance, but post-match fatigue monitoring should be strengthened. In terms of halftime scheduling, a moderate-intensity RWU of approximately 3 min with appropriate active recovery may be prioritised. However, given the limitations of the current evidence, the decision to include or omit a brief interval after RWU should be made cautiously and should take into account competition timing, venue conditions, and athlete readiness.

Particular attention should be paid to monitoring RPE and performance responses in adolescent athletes, who showed larger RPE increases in the available studies. In practice, coaches should monitor both subjective and objective responses during and after RWU. Subjective indicators, such as RPE, should be combined with objective performance measures, such as CMJ height, sprint time, or other sport-specific performance indicators, to avoid relying on rating of perceived exertion alone when making training decisions.

Although warm-up exercise type was not a significant moderator, specific protocols produced numerically larger estimates for some outcomes. This trend may have a neurophysiological basis. Specific warm-ups may increase tissue temperature, reduce muscle and joint viscosity, improve contraction efficiency, accelerate neural impulse transmission, and enhance central nervous system function and reaction time. Accordingly, coaches may consider including warm-up exercises that are closely related to the movement patterns and performance demands of the target sport. However, the additional benefit of specific warm-ups over general warm-ups still requires confirmation through larger randomised controlled trials.

### 4.3. Potential limitations and future directions

Although this meta-analysis provides a comprehensive evaluation of the effects of RWU strategies on performance enhancement and rating of perceived exertion in team-sport athletes, several limitations should be acknowledged. First, some of the included studies were conducted under simulated team-sport conditions rather than real competitive settings. In several studies, participants performed cycle ergometer protocols or intermittent cycling tasks to simulate the metabolic demands of team sports, rather than completing sport-specific technical actions. This may limit external validity, because RWU in real competition needs to integrate technical–tactical preparation with physiological and metabolic activation. To examine whether competition setting contributed to heterogeneity, we compared simulated and real team-sport conditions. Subgroup analysis showed that the direction of RWU effects was consistent across these conditions, with no significant interaction effect (p > 0.05) (see S13 File).

Second, although potential moderators such as training age and environmental temperature were collected during data extraction, these variables were incompletely reported in the original studies. As a result, we were unable to fully examine the associations of training age and environmental temperature with intervention effects. Because these variables were not critical for calculating the main effect sizes, studies with missing information on these variables were retained in the analysis. Nevertheless, this missing information limited our ability to explore how training background and environmental conditions may influence RWU effects, and should therefore be regarded as an important limitation of the present study.

Third, to avoid excessive complexity in the multivariable analyses, not all possible variable combinations were tested. This may have led to the omission of some potentially meaningful interaction effects. Instead, we prioritised combined tests for variables that were statistically significant in univariate analyses or theoretically relevant. In addition, to explore sources of heterogeneity and the boundary conditions of RWU effects, this study conducted multidimensional univariate moderator analyses, multivariable interaction analyses, and nonlinear meta-regression analyses. However, some outcomes, particularly maximal strength, were based on a small number of included studies. Therefore, the risks of model overfitting and false-positive findings cannot be fully eliminated. Future research should include more high-quality primary trials to strengthen the evidence base for these outcomes.

Fourth, to reduce language bias as much as possible, the search strategy included CNKI. However, after eligibility screening, no Chinese-language studies met the inclusion criteria; consequently, the current evidence base provides limited information on the generalisability of RWU effects to Chinese athlete populations and research settings.

## 5. Conclusion

Compared with passive rest, RWU may improve countermovement jump (CMJ) height, sprint speed, and agility in team-sport athletes. However, several subgroup and interaction findings require confirmation in larger and more diverse evidence bases because many analyses included relatively few studies or effect sizes. RWU did not significantly improve squat jump (SJ) height, repeated sprint ability, or maximal strength, and it may increase rating of perceived exertion under certain conditions.

For CMJ performance, preliminary findings suggest that RWU effects may be moderated by takeoff style, with unilateral takeoff showing greater effects than bilateral takeoff, and by lower-limb dominance, with the dominant limb showing greater effects than the non-dominant limb. Significant interactions among takeoff style, lower-limb dominance, interval format, and age group were observed under specific conditions. Exploratory interaction analyses suggested larger CMJ effect estimates for RWU without a post-RWU interval, for unilateral versus bilateral jumps, and for the dominant versus non-dominant limb; larger effects were also observed in adolescent athletes in some models. However, the mechanisms underlying these moderator effects require further investigation.

For sprint performance, interval format appeared to influence the effect of RWU on sprint time, with RWU including an interval showing greater benefits than RWU without an interval. Significant interactions were also observed among running distance, interval format, and age group. Specifically, when any two variables were controlled for, greater effects tended to appear among adolescent athletes, under RWU with intervals, and over 5–10 m sprint distances. In the available subgroup analyses, RWU with a post-RWU interval was associated with better sprint performance than passive rest across the examined age groups and sprint distances. By contrast, under RWU without an interval, only 5–20 m sprint performance in adolescent athletes was significantly better than passive rest in the included analyses.

For agility performance, no significant moderators were identified. In some subgroup analyses, RWU was associated with better agility performance than passive rest only among adolescent athletes who performed RWU without a post-RWU interval. For rating of perceived exertion, potential interactions were observed among age group, effect category, and interval format. Adolescent athletes showed greater changes in rating of perceived exertion than adults who performed RWU and passive-rest controls both immediately after RWU and during the 5–45 min period of the second half, regardless of whether rest was provided after RWU. Adult athletes showed increased rating of perceived exertion only immediately after RWU compared with passive rest.

Overall, the optimal implementation of RWU is likely to depend on multiple contextual factors, including athlete age, training level, sport-specific demands, competition environment, and individual physiological differences. Because the current evidence is not yet sufficient to establish a universal optimal RWU protocol, coaches should individualise RWU strategies based on existing evidence, professional judgement, and athlete monitoring, while continuing to follow emerging research in this field.

## Supporting information

S1 File Search strategy. (DOCX)

S2 File Reference list of included studies. (DOCX)

S3 File Characteristics of included studies and participants, intervention protocols, and outcome measures. (DOCX)

S4 File Risk of bias assessment of the included studies. (DOCX)

S5 File Publication bias analyses for the primary outcomes. (DOCX)

S6 File GRADE evidence profiles and certainty assessments for the primary outcomes. (DOCX)

S7 File Leave-one-out sensitivity analyses for the primary outcomes. (DOCX)

S8 File Results of traditional two-level meta-analyses and corresponding forest plots. (DOCX)

S9 File Moderator analyses, including continuous-variable analyses, RWU exercise-type analyses, and multivariable moderator analyses. (DOCX)

S10 File Sensitivity analyses using different assumed correlation coefficients. (DOCX)

S11 File Detailed re-warm up (RWU) protocol extraction table and extraction-consensus records. (DOCX)

S12 File Subgroup analyses and comparisons across different sports. (DOCX)

S13 File Comparative analyses of simulated and real competitive environments. (DOCX)
