# Supplementary material for: The effect of re-warm up and its moderators on performance enhancement and rating of perceived exertion in team-sport athletes: A systematic review and three-level meta-analysis
Source: PLoS One. 2026 Sep 18;21(9):e0358479. doi: 10.1371/journal.pone.0358479 (PMC13588333; doi:10.1371/journal.pone.0358479)
Supplement: Supplementary file 1 [file pone.0358479.s001.docx]

**S1 File. Search Strategy**

Search execution period: April–June 2025

Coverage period: From database inception to 30 June 2025.

Search conducted by: HL-T (all databases)

Search verification: The search strategy was developed by HLT and independently checked by JZ. PubMed and Web of Science searches were replicated by JZ to verify syntax accuracy and reproducibility.

**Database 1: PubMed**

**Limits: None**

| Query | Number |
| --- | --- |
| ((("re-warm up"[Title/Abstract] OR "rewarm up"[Title/Abstract] OR "re warm up"[Title/Abstract] OR "post-warm up"[Title/Abstract] OR "post warm up"[Title/Abstract] OR "halftime warm up"[Title/Abstract] OR "mid-interval warm up"[Title/Abstract] OR "half-time warm up"[Title/Abstract]) OR ("Warm-Up Exercise"[Mesh] AND ("halftime"[Title/Abstract] OR "half time"[Title/Abstract] OR "mid-interval"[Title/Abstract]))) AND (("athletic performance"[Title/Abstract] OR "sport performance"[Title/Abstract] OR "sprint performance"[Title/Abstract] OR "jump performance"[Title/Abstract] OR "agility test"[Title/Abstract] OR "change of direction"[Title/Abstract] OR "COD"[Title/Abstract]) OR ("sprint"[Title/Abstract] OR "sprinting"[Title/Abstract] OR "countermovement jump"[Title/Abstract] OR "CMJ"[Title/Abstract] OR "squat jump"[Title/Abstract] OR "SJ"[Title/Abstract] OR "power output"[Title/Abstract] OR "muscle power"[Mesh])) | 85 |

#### ****Database 2: Web of Science****

****Search fields:**** TS = Topic (Title, Abstract, Author Keywords, Keywords Plus)
****Limits:**** None

| Query | Number |
| --- | --- |
| TS=(("re-warm up" OR "rewarm up" OR "re warm up" OR "post-warm up" OR "post warm up" OR "halftime warm up" OR "mid-interval warm up" OR "half-time warm up") OR ("rewarmup" OR "rewarm-up" OR "postwarmup" OR "halftime warm-up")  AND  TS=("athletic performance" OR "sprint performance" OR "jump height" OR "agility test" OR "change of direction" OR "COD test" OR "power output") OR ("sprint time" OR "countermovement jump" OR "CMJ" OR "squat jump" OR "SJ" OR "repeated sprint ability") | 25 |

#### ****Database 3: Springer Link****

****Filters applied:**** Content type: Journal Articles; Discipline: Sports Science; Language: English

| Query | Number |
| --- | --- |
| ("re-warm up" OR "rewarm up" OR "re warm up" OR "post-warm up" OR "post warm up" OR "halftime warm up" OR "mid-interval warm up" OR "rewarmup" OR "rewarm-up" OR "postwarmup" OR "halftime warm-up")  AND  ("performance" OR "sprint performance" OR "jump height" OR "agility test" OR "change of direction" OR "COD test" OR "power output" OR "sprint time" OR "countermovement jump" OR "CMJ" OR "squat jump" OR "SJ" OR "repeated sprint ability") | 142 |

#### ****Database 4: ProQuest****

****Search fields:**** NOFT = NO Full Text (searches title, abstract, subject headings only)
****Filters applied:**** Source type: Scholarly Journals; Document type: Article

| Query | Number |
| --- | --- |
| noft ("re-warm up" OR "rewarm up" OR "re warm up" OR "post-warm up" OR "post warm up" OR "halftime warm up" OR "mid-interval warm up" OR "rewarmup" OR "rewarm-up" OR "postwarmup" OR "halftime warm-up")  AND  noft ("athletic performance") OR "sprint performance" OR "jump height" OR "agility test" OR "change of direction" OR "COD test" OR "power output" OR "sprint time" OR "countermovement jump" OR "CMJ" OR "squat jump" OR "SJ" OR "repeated sprint ability") | 21 |

#### ****Database 5: SPORTDiscus****

****Search fields:**** TI = Title (initial search); expanded to TI+AB (Title + Abstract) after preliminary screening
****Filters applied:**** Publication type: Journal Article; Peer-reviewed

| Query | Number |
| --- | --- |
| TI ("re-warm up" OR "rewarm up" OR "re warm up" OR "post-warm up" OR "post warm up" OR "halftime warm up" OR "mid-interval warm up" OR "half-time warm up" OR "rewarmup" OR "rewarm-up" OR "postwarmup" OR "halftime warm-up")  AND  TI ("performance" OR "sprint performance" OR "jump height" OR "agility test" OR "change of direction" OR "COD test" OR "power output" OR "sprint time" OR "countermovement jump" OR "CMJ" OR "squat jump" OR "SJ" OR "repeated sprint ability") | 108 |

#### ****Database 6: CNKI****

****Search fields:**** SU = Subject
****Filters applied:**** Source type: Academic Journals; Discipline: Physical Education and Sports Science

| Query | Number |
| --- | --- |
| SU=('热身(warm up)' OR '二次热身(re-warm up)' OR '再热身'("Re Warm Up") OR '中场热身(halftime warm up )' OR '半场热身' (mid-interval warm up) AND SU=('运动表现' (sport performance)+ '竞技表现'(competition performance) + '运动状态'(athletic performance) + '力量'(power output) + '速度'(agility test) + '耐力'(repeated sprint ability) | 657 |
