# Supplementary material for: The effect of re-warm up and its moderators on performance enhancement and rating of perceived exertion in team-sport athletes: A systematic review and three-level meta-analysis
Source: PLoS One. 2026 Sep 18;21(9):e0358479. doi: 10.1371/journal.pone.0358479 (PMC13588333; doi:10.1371/journal.pone.0358479)
Supplement: Supplementary file 2 [file pone.0358479.s002.docx]

**S2 File.Reference list of included studies**

[1] Yang YR, Chen C, Pan CH, Yen SY, Cheng CF. Effects of half-time re-warm-up with core strength exercises on subsequent agility and power performance in basketball players. J Strength Cond Res. 2025;39(1):62-69. doi: 10.1519/JSC.0000000000004948.
[2] Mohr M, Krustrup P, Nybo L, Nielsen JJ, Bangsbo J. Muscle temperature and sprint performance during soccer matches—beneficial effect of re-warm-up at half-time. Scand J Med Sci Sports. 2004;14(3):156-162. doi: 10.1111/j.1600-0838.2004.00349.x.
[3] Edholm P, Krustrup P, Randers MB. Half-time re-warm up increases performance capacity in male elite soccer players. Scand J Med Sci Sports. 2014. doi: 10.1111/sms.12236.
[4] Lovell R, Midgley A, Barrett S, Carter D, Small K. Effects of different half-time strategies on second half soccer-specific speed, power and dynamic strength. Scand J Med Sci Sports. 2013;23(1):105-113. doi: 10.1111/j.1600-0838.2011.01353.x.
[5] Yanaoka T, Kashiwabara K, Masuda Y, Kurata K, Yamamoto R, Miyashita M, et al. The effect of halftime re-warm up duration on intermittent sprint performance. J Sports Sci Med. 2018;17(2):269.
[6] Christaras M, Michailidis Y, Mandroukas A, Vardakis L, Christoulas K, Metaxas T. Effects of a short halftime re-warm-up program on matches running performance and fitness test performance of male elite youth soccer players. Appl Sci. 2023;13(4):2602. doi: 10.3390/app13042602.
[7] Koutsouridis C, Galazoulas C, Manou V, Stavropoulos N, Bassa E. Evaluating the impact of a 3-min moderate-intensity re-warm-up protocol on basketball player performance. J Phys Educ Sport. 2024;24(5):1099-1108. doi:10.7752/jpes.2024.05126.
[8] Fashioni E, Langley B, Page RM. The effectiveness of a practical half-time re-warm-up strategy on performance and the physical response to soccer-specific activity. J Sports Sci. 2020 Jan;38(2):140-149. doi: 10.1080/02640414.2019.1686941.
[9] Ramos-Campo DJ, Malta J, Olcina G, Timón R, Raimundo A, Tomas-Carus P. Impact of active and passive hypoxia as re-warm-up activities on rugby players' performance. Int J Environ Res Public Health. 2020;17(8):2971. doi: 10.3390/ijerph17082971.
[10] Ltifi MA, Turki O, Racil G, Larion A, Chelly MS, Ben Saad H, et al. A 3-min weighted vests re-warmups induce sprint performance enhancements at the start of the second half of a soccer match-play. Front Physiol. 2023;14:1173991. doi: 10.3389/fphys.2023.1173991.
[11] Matsentides D, Christou M, Zaras N. The effects of different re-warm-up strategies on power, changing of direction and ball shooting velocity in well-trained soccer players. Sports (Basel). 2023;11(9):169. doi: 10.3390/sports11090169.
[12] Sanchez-Sanchez J, Clemente FM, Ramirez-Campillo R, Rodríguez-Fernández A. Elastic bands during half-squats as a re-warm-up strategy for youth soccer players' performance. J Sports Sci Med. 2024;23(4):843. doi: 10.52082/jssm.2024.843.
[13] Tong TK, Baker JS, Zhang H, Kong Z, Nie J. Effects of specific core re-warm-ups on core function, leg perfusion and second-half team sport-specific sprint performance: a randomized crossover study. J Sports Sci Med. 2019;18(3):479-489. PMID: 31427870.
[14] Yamashita Y, Umemura Y. Effect of High-Intensity, Intermittent, Short-Duration Re-Warming up on Cycling Sprint Performance. J Hum Kinet. 2022 Sep 8;83:131-141. doi: 10.2478/hukin-2022-0068.
[15] Yanaoka T, Hamada Y, Kashiwabara K, Kurata K, Yamamoto R, Miyashita M, et al. Very-short-duration, low-intensity halftime re-warm up increases subsequent intermittent sprint performance. J Strength Cond Res. 2018 Nov;32(11):3258-3266. doi: 10.1519/JSC.0000000000002781.
[16] Yanaoka T, Iwata R, Yoshimura A, Hirose N. A 1-minute re-warm up at high-intensity improves sprint performance during the Loughborough intermittent shuttle test. Front Physiol. 2021;11:616158. doi: 10.3389/fphys.2020.616158.
[17] Yanaoka T, Hamada Y, Fujihira K, Yamamoto R, Iwata R, Miyashita M, et al. High-intensity cycling re-warm up within a very short time-frame increases the subsequent intermittent sprint performance. Eur J Sport Sci. 2020 Nov;20(10):1307-1317. doi: 10.1080/17461391.2020.1713901.
[18] González-Devesa D, Vaquera A, Suárez-Iglesias D, Ayán C. Effects of halftime re-warm-up implemented during a simulated match in U14 female basketball players. J Sports Sci. 2022 Dec;40(23):2681-2687. doi: 10.1080/02640414.2023.2184528.
[19] Flórez-Gil E, Mateus N, Sampaio J, Abade E. Examining the impact of different re-warm-up strategies on non-starter basketball players' physical performance. J Sports Sci. 2025 Mar;43(6):536-543. doi: 10.1080/02640414.2025.2467564.
[20] Zois J, Bishop D, Fairweather I, Ball K, Aughey RJ. High-intensity re-warm-ups enhance soccer performance. Int J Sports Med. 2013 Sep;34(9):800-5. doi: 10.1055/s-0032-1331197.
