# Supplementary material for: The effect of re-warm up and its moderators on performance enhancement and rating of perceived exertion in team-sport athletes: A systematic review and three-level meta-analysis
Source: PLoS One. 2026 Sep 18;21(9):e0358479. doi: 10.1371/journal.pone.0358479 (PMC13588333; doi:10.1371/journal.pone.0358479)
Supplement: Supplementary file 3 [file pone.0358479.s003.docx]

**S3 File. Characteristics of included studies and participants, intervention protocols, and outcome measures.**

| **Author** | **Characteristics of the subject** | | | |  | **Interventions information** | | | | |  | **outcome** | |
| --- | --- | --- | --- | --- | --- | --- | --- | --- | --- | --- | --- | --- | --- |
|  | **Number** | **age (mean[SD])** | **BMI (mean)** | **sports** | **intensity** | | **RWU strategy** | **volume (min/session)** | **Mode of organisation** | **Intervals**  **（With or without）** | | **outcomes** | **Measurement method and unit** |
| Christaras M  2023 | 23 | 16.5 (0.4) | 22.95 | football | H | | Running at 70–90% of max speed for 30 m； Skipping for 20 m；3 jumps with knees to chest and sprint 30 m； Sprint and change of direction 180◦ every 5 m (total 20 m)； Reps1， Rest~30s | 3 min | 12min rest+3 min RWU | Without Intervals | | Speed、Jumps、Agility | 10m\30m\CMJ\SJ\Illinois test |
| P.Edholm  2014 | 17 | 25  （18-33 years） | 23.73 | football | ML | | 7 min of low-/moderate intensity jogging and light calisthenics at ∼70% of maximal heart rate | 7 min | 7min rest +7 min RW | With Interval | | Jumps  Speed | CMJ  10m |
| Christos Koutsouridis  2024 | 13 | 20.54(1.45) | 24.34 | basketball | ML | | 3 min cycling at 40% VО_z_max | 3 min | 11min rest +3 min RW | Without Intervals | | Jumps  Agility  RPE | CMJ  Modified Agility T-tes  RPE（immediately） |
| Fashioni  2020 | 10 | 23（4） | 24.34 | football | H | | The participants were instructed to perform two sets of jogging (40 m) and skipping (40 m), followed by one set (20 m) of each dynamic stretch exercise. Following the completion of the aforementioned dynamic stretches, participants completed two sets each of straight sprinting, tuck jump into sprints, and reaction sprints. | 3 min | 12min rest + 3 min RW | Without Intervals | | Speed  Jumps  RPE | 5 m\10m\20m  CMJ\SJ  RPE（immediately or after 10-20 min） |
| Ramos-Campo  2020 | 10 | 22.9（5.3） | 27.47 | rugby | MH | | 4x5 maximum horizontal jumps and 15s of passive recovery between sets | 4.5 min | 7.5min rest + 4.5 min RW+ 3min rest | With Interval | | Speed  Jumps  RPE  RSA(mean)  RSA(peak) | 20m  CMJ  RPE（immediately）  RSA（hypoxia）  RSA（normoxia） |
| Lovell  2011 | 10 | 20(1.0) | 23.86 | football | M | | Intermittent Agility Exercise or Whole Body Vibration | 5min | 9 min rest + 5min RW + 1min rest | With Interval | | Speed  Jumps  strength | 10m  CMJ  Eccentric hamstring（peak torque） |
| Ltif  2023 | 20 | 14.55(0.51) | 20.45 | football | H | | 3 x 15m sprint（with a  weighted vest loaded at 0%, 5%,10% BM） | 3min | 10 min rest+3min RW + 2min rest | With Interval | | Speed  RPE | 20m  RPE（immediately） |
| Matsentides  2023 | 10 | 18.2 (1.7) | 22.02 | football | H | | JS: 4 maximum tuck jumps and then a maximum 20 m linear sprint. Players performed 4 repetitions with 40 s rest between repetitions.  COD: 4 repetitions of maximum velocity sprints with 180° change of direction every 5 m covering a 20 m total distance with 40 s rest between repetitions. Players were instructed to sprint and accelerate, and decelerate as fast as possible | 4min | 8 min rest + 4 RW+ 3min rest | With Interval | | Jumps  Agility | CMJ  T-Test |
| Mohr  2004 | 16 | EG：26.07(0.5)  CG：25.87(1.4) | 23.10 | football | M | | running and other exercises at a moderate intensity | 7min | 7 min rest+7min RW+1 min rest | With Interval | | Speed | 30m |
| Javier Sanchez-Sanchez  2024 | 20 | 15.7 (0.8) | 23.46 | football | ML | | SQ：half-squat without elastic looped band  SQT：half-squat with elastic looped band around the thigh  SQL：half-squat with elastic looped band around the lower leg | 5min | 10 min rest + 5 minRW | Without Intervals | | Vertical Jumps\horizontal jumps\speed\agility | SJ\SJd\SJnd\CMJ\CMJd\CMJnd\Hop jump distance\triple hop jump distance\20m\T-test |
| Tomas K. Tong  2019 | 9 | 20.6(0.9) | 22.72 | football/handball | ML | | inspiratory-loaded core muscle training | 4min | 11min rest + 4min RW | Without Intervals | | RSA  RPE  blood lactate | RSA  RPE（immediately or during Comp）  [La] |
| Yamashita  2022 | 10 | 21 (2.0) | 22.02 | set in a team sport | H | | high-intensity intermittent cycling | 2min | 12 min rest + 2min RW+1 min rest | With Interval | | blood lactate  RPE | [La]  RPE（immediately or after 10 min） |
| Yanaoka  2018 | 13 | 22.4( 2.1) | 22.65 | set in a team sport | M | | 7 min RW: 7min cycling at70% of HR max  3 min RW: 3 min of cycling at 70% of HR max | 7min | 7min rest + 7min RW + 1min rest  11min + 3min RW + 1 min rest | With Interval | | RPE | RPE（immediately or after 10 min） |
| Yanaoka  2018 | 11 | 22.7( 2.4) | 21.82 | set in a team sport | M | | cycling at 30% Vо_2_ max or cycling at 60% VО_2_ max | 3min | 11min rest +3 min RW（60% or 30%）+ 1 min rest | With Interval | | RPE | RPE（immediately or after 10 -20 min） |
| Yanaoka  2021 | 12 | 22(2.0） | 22.40 | team sport player | H | | 1 minute of high-intensity running (90% VO₂ max, speed 261 ± 19 m/min) | 1min | 14min rest +1min RW | With Interval | | RPE | RPE（immediately or after 45 min） |
| Yanaoka  2020 | 12 | 23（2.0） | 23.43 | set in a team sport | L or H | | 3 min RW：cycling at 30% of VO_2_max；  1 min RW cycling at 90% of VO_2_max | 3min | 11 min rest +3 min RW+1min rest；  13 min rest + 1 min RW +1 min rest | With Interval | | RPE  Speed | RPE（immediately）  sprint power |
| González-Devesa  2023 | 10 | 13.7  （0.8） | 20.85 | basketball | H | | a combination of maximal intensity sprints (5×14m) for 1 min and a shooting wheel for 2 min | 3min | 6min rest +3 min RW+1min rest | With Interval | | Jumps  RPE | CMJ  RPE（immediately） |
| Flórez-Gil  2025 | 15 | 16.7  （0.6） | 21.83 | basketball | ML | | Plyometric exercise or dynamic stretching | 1min | A structured cycle that consists of 2 min of rest and 1 min of RW | Without Intervals | | Jumps  Agility | CMJ  DJ  COD |
| Yang  2024 | 12 | 20.5  （1.6） | 24.28 | basketball | ML | | STA：Core Strength on a stable platform condition  USTA： Core Strength on an unstable platform condition | 4minR | 10 min rest+4 min RW+1 min rest | With Interval | | blood lactate  RPE  Agility  Jumps  strength | La  RPE（immediately）  T-Test for COD  CMJ  SJ  Maximal strength  RFD max  RFD30-250 |
| J. Zois   2012 | 8 | 23.6  （4.1） | 25.23 | football | H | | 5RM Leg Press | 15s | 10min rest +15s+ 4min rest | With Interval | | RSA | Peak velocity  Mean velocity |

**Annotation：H:** high intensity; **M:** moderate intensity; **L:** low intensity. **CMJ:** Countermovement Jump; **SJ:** Squat Jump; **RSA:** Repeated Sprint Ability; **RPE:** rating of perceived exertion; **SJd:** SJ with dominant leg; **SJnd:** SJ with non-dominant leg; **CMJd:** CMJ with dominant leg; **CMJnd:** CMJ with non-dominant leg; **DJ:** Drop Jump; **COD:** Change of Direction Speed; **RFD:** Rate of Force Development; **Set in a team sport:** exercise programmes that simulate the characteristics of team sports like football
