# Supplementary material for: The effect of re-warm up and its moderators on performance enhancement and rating of perceived exertion in team-sport athletes: A systematic review and three-level meta-analysis
Source: PLoS One. 2026 Sep 18;21(9):e0358479. doi: 10.1371/journal.pone.0358479 (PMC13588333; doi:10.1371/journal.pone.0358479)
Supplement: Supplementary file 6 [file pone.0358479.s006.docx]

**S6 File. GRADE evidence profiles and certainty assessments for the primary outcomes.**

| **Performance** | **Study limitations** | **Imprecision** | **Inconsistency** | **Indirectness** | **Publication**  **bias** | **GRADE** |
| --- | --- | --- | --- | --- | --- | --- |
| **CMJ** | No downgrade | Downgrade -1  (because point estimate＜1.0 but lower limit ＞1.25） | No downgrade | No downgrade | No downgrade | Moderate |
| **SJ** | No downgrade | Downgrade -1  (because point estimate＜1.0 but lower limit ＞1.25） | No downgrade | No downgrade | No downgrade | Moderate |
| **Sprint time** | No downgrade | No downgrade | Downgrade -1  (because of heterogeneity） | No downgrade | No downgrade | Moderate |
| **RAS** | No downgrade | No downgrade | No downgrade | No downgrade | Downgrade-1  (because of publication bias） | Moderate |
| **Agility** | No downgrade | No downgrade | No downgrade | No downgrade | No downgrade | High |
| **Max strength** | No downgrade | Downgrade -2 | No downgrade | No downgrade | No downgrade | Low |
| **RPE** | No downgrade | Downgrade -1  (because point estimate＜1.0 but lower limit ＜0.38） | Downgrade -1  （because of heterogeneity） | No downgrade | No downgrade | Low |
