# Supplementary material for: The effect of re-warm up and its moderators on performance enhancement and rating of perceived exertion in team-sport athletes: A systematic review and three-level meta-analysis
Source: PLoS One. 2026 Sep 18;21(9):e0358479. doi: 10.1371/journal.pone.0358479 (PMC13588333; doi:10.1371/journal.pone.0358479)
Supplement: Supplementary file 7 [file pone.0358479.s007.docx]

**S7 File. Leave-one-out sensitivity analyses for the primary outcomes.**

**Table 1 Results of sensitivity analysis of RSA**

| **Excluded_ID** | **Estimate** | **CI_LB** | **CI_UB** | **p_Value** | **Significance** |
| --- | --- | --- | --- | --- | --- |
| **1** | -0.38 | -0.82 | 0.06 | 0.08 | Non-Sig |
| **2** | -0.36 | -0.80 | 0.08 | 0.09 | Non-Sig |
| **3** | -0.38 | -0.81 | 0.06 | 0.08 | Non-Sig |
| **4** | -0.37 | -0.80 | 0.07 | 0.09 | Non-Sig |
| **5** | -0.32 | -0.76 | 0.12 | 0.13 | Non-Sig |
| **6** | -0.30 | -0.73 | 0.13 | 0.14 | Non-Sig |
| **7** | -0.32 | -0.76 | 0.12 | 0.13 | Non-Sig |
| **8** | -0.31 | -0.74 | 0.12 | 0.13 | Non-Sig |

The results showed that after excluding any of the studies in the RSA, the overall effect value changed only slightly and remained statistically insignificant (p > 0.05).

**Table 2 Results of sensitivity analysis of CMJ**

| **Excluded_ID** | **Estimate** | **CI_LB** | **CI_UB** | **p_Value** | **Significance** |
| --- | --- | --- | --- | --- | --- |
| **1** | 0.86 | 0.30 | 1.42 | 0.00 | Sig |
| **2** | 0.85 | 0.28 | 1.41 | 0.00 | Sig |
| **3** | 0.84 | 0.28 | 1.40 | 0.01 | Sig |
| **4** | 0.83 | 0.27 | 1.39 | 0.01 | Sig |
| **5** | 0.85 | 0.31 | 1.39 | 0.00 | Sig |
| **6** | 0.83 | 0.28 | 1.37 | 0.00 | Sig |
| **7** | 0.82 | 0.28 | 1.37 | 0.00 | Sig |
| **8** | 0.81 | 0.27 | 1.35 | 0.01 | Sig |
| **9** | 0.84 | 0.30 | 1.38 | 0.00 | Sig |
| **10** | 0.84 | 0.30 | 1.38 | 0.00 | Sig |
| **11** | 0.81 | 0.30 | 1.33 | 0.00 | Sig |
| **12** | 0.83 | 0.29 | 1.37 | 0.00 | Sig |
| **13** | 0.82 | 0.29 | 1.34 | 0.00 | Sig |
| **14** | 0.78 | 0.31 | 1.24 | 0.00 | Sig |
| **15** | 0.83 | 0.29 | 1.37 | 0.00 | Sig |
| **16** | 0.80 | 0.30 | 1.31 | 0.00 | Sig |
| **17** | 0.82 | 0.29 | 1.35 | 0.00 | Sig |
| **18** | 0.83 | 0.29 | 1.37 | 0.00 | Sig |
| **19** | 0.80 | 0.30 | 1.30 | 0.00 | Sig |
| **20** | 0.71 | 0.20 | 1.23 | 0.01 | Sig |
| **21** | 0.81 | 0.27 | 1.35 | 0.01 | Sig |
| **22** | 0.84 | 0.31 | 1.38 | 0.00 | Sig |
| **23** | 0.84 | 0.31 | 1.37 | 0.00 | Sig |
| **24** | 0.84 | 0.30 | 1.38 | 0.00 | Sig |
| **25** | 0.84 | 0.30 | 1.38 | 0.00 | Sig |

The results showed that after excluding any of the studies in the CMJ, the overall effect value changed only slightly and was statistically significant (p＜0.05).

**Table 3 Results of sensitivity analysis of SJ**

| **Excluded_ID** | **Estimate** | **CI_LB** | **CI_UB** | **p_Value** | **Significance** |
| --- | --- | --- | --- | --- | --- |
| **1** | 0.83 | -0.09 | 1.74 | 0.07 | Non-Sig |
| **2** | 0.76 | -0.18 | 1.70 | 0.10 | Non-Sig |
| **3** | 0.65 | -0.03 | 1.32 | 0.06 | Non-Sig |
| **4** | 0.72 | -0.07 | 1.50 | 0.07 | Non-Sig |
| **5** | 0.72 | -0.10 | 1.54 | 0.08 | Non-Sig |
| **6** | 0.68 | -0.04 | 1.40 | 0.06 | Non-Sig |
| **7** | 0.71 | -0.06 | 1.48 | 0.07 | Non-Sig |
| **8** | 0.71 | -0.11 | 1.53 | 0.08 | Non-Sig |
| **9** | 0.71 | -0.06 | 1.49 | 0.07 | Non-Sig |
| **10** | 0.72 | -0.09 | 1.54 | 0.08 | Non-Sig |
| **11** | 0.73 | -0.09 | 1.54 | 0.08 | Non-Sig |
| **12** | 0.71 | -0.14 | 1.56 | 0.09 | Non-Sig |
| **13** | 0.81 | 0.02 | 1.59 | 0.05 | Sig |

In the SJ, when the indicators from the USTA group in Yang et al.'s study (2024) were excluded, the pooled effect size changed from non-significant to significant (g = 0.81, P = 0.05).

**Table 4 Results of sensitivity analysis of sprint time**

| **Excluded_ID** | **Estimate** | **CI_LB** | **CI_UB** | **p_Value** | **Significance** |
| --- | --- | --- | --- | --- | --- |
| **1** | -0.79 | -1.24 | -0.33 | 0.00 | Sig |
| **2** | -0.87 | -1.30 | -0.45 | 0.00 | Sig |
| **3** | -0.80 | -1.28 | -0.32 | 0.00 | Sig |
| **4** | -0.82 | -1.23 | -0.41 | 0.00 | Sig |
| **5** | -0.83 | -1.22 | -0.44 | 0.00 | Sig |
| **6** | -0.82 | -1.24 | -0.40 | 0.00 | Sig |
| **7** | -0.84 | -1.27 | -0.41 | 0.00 | Sig |
| **8** | -0.82 | -1.25 | -0.38 | 0.00 | Sig |
| **9** | -0.82 | -1.26 | -0.39 | 0.00 | Sig |
| **10** | -0.82 | -1.25 | -0.39 | 0.00 | Sig |
| **11** | -0.83 | -1.26 | -0.40 | 0.00 | Sig |
| **12** | -0.83 | -1.25 | -0.40 | 0.00 | Sig |
| **13** | -0.77 | -1.15 | -0.40 | 0.00 | Sig |
| **14** | -0.71 | -1.06 | -0.35 | 0.00 | Sig |
| **15** | -0.81 | -1.24 | -0.37 | 0.00 | Sig |
| **16** | -0.82 | -1.24 | -0.40 | 0.00 | Sig |
| **17** | -0.84 | -1.24 | -0.43 | 0.00 | Sig |

The results showed that after excluding any of the studies in the sprint time, the overall effect value changed only slightly and was statistically significant (p＜0.05).

**Table 5 Results of sensitivity analysis of agility**

| **Excluded_ID** | **Estimate** | **CI_LB** | **CI_UB** | **p_Value** | **Significance** |
| --- | --- | --- | --- | --- | --- |
| **1** | -0.55 | -0.91 | -0.19 | 0.01 | Sig |
| **2** | -0.49 | -0.87 | -0.11 | 0.02 | Sig |
| **3** | -0.50 | -0.85 | -0.16 | 0.01 | Sig |
| **4** | -0.49 | -0.85 | -0.14 | 0.01 | Sig |
| **5** | -0.43 | -0.68 | -0.18 | 0.00 | Sig |
| **6** | -0.46 | -0.77 | -0.15 | 0.01 | Sig |
| **7** | -0.52 | -0.93 | -0.10 | 0.02 | Sig |
| **8** | -0.49 | -0.84 | -0.15 | 0.01 | Sig |
| **9** | -0.48 | -0.83 | -0.13 | 0.01 | Sig |
| **10** | -0.50 | -0.84 | -0.16 | 0.01 | Sig |
| **11** | -0.46 | -0.81 | -0.10 | 0.02 | Sig |
| **12** | -0.49 | -0.85 | -0.13 | 0.01 | Sig |

The results showed that after excluding any of the studies in the agility, the overall effect value changed only slightly and was statistically significant (p＜0.05).

**Table 6 Results of sensitivity analysis of RPE**

| **Excluded_ID** | **Estimate** | **CI_LB** | **CI_UB** | **p_Value** | **Significance** |
| --- | --- | --- | --- | --- | --- |
| **1** | 1.05 | 0.32 | 1.77 | 0.01 | Sig |
| **2** | 1.04 | 0.37 | 1.71 | 0.00 | Sig |
| **3** | 1.04 | 0.37 | 1.71 | 0.00 | Sig |
| **4** | 1.07 | 0.40 | 1.74 | 0.00 | Sig |
| **5** | 1.06 | 0.39 | 1.73 | 0.00 | Sig |
| **6** | 1.05 | 0.38 | 1.73 | 0.00 | Sig |
| **7** | 1.10 | 0.32 | 1.89 | 0.01 | Sig |
| **8** | 0.99 | 0.42 | 1.56 | 0.00 | Sig |
| **9** | 1.00 | 0.42 | 1.58 | 0.00 | Sig |
| **10** | 1.06 | 0.39 | 1.74 | 0.00 | Sig |
| **11** | 1.04 | 0.37 | 1.72 | 0.00 | Sig |
| **12** | 0.98 | 0.30 | 1.65 | 0.01 | Sig |
| **13** | 1.11 | 0.45 | 1.78 | 0.00 | Sig |
| **14** | 1.02 | 0.36 | 1.69 | 0.00 | Sig |
| **15** | 1.08 | 0.41 | 1.75 | 0.00 | Sig |
| **16** | 1.03 | 0.36 | 1.69 | 0.00 | Sig |
| **17** | 1.03 | 0.36 | 1.70 | 0.00 | Sig |
| **18** | 1.05 | 0.38 | 1.72 | 0.00 | Sig |
| **19** | 1.05 | 0.38 | 1.72 | 0.00 | Sig |
| **20** | 1.05 | 0.38 | 1.72 | 0.00 | Sig |
| **21** | 1.05 | 0.39 | 1.72 | 0.00 | Sig |
| **22** | 1.05 | 0.38 | 1.72 | 0.00 | Sig |
| **23** | 1.02 | 0.35 | 1.70 | 0.00 | Sig |
| **24** | 1.07 | 0.40 | 1.75 | 0.00 | Sig |
| **25** | 1.07 | 0.40 | 1.74 | 0.00 | Sig |
| **26** | 1.03 | 0.35 | 1.71 | 0.00 | Sig |
| **27** | 1.04 | 0.32 | 1.76 | 0.01 | Sig |
| **28** | 1.08 | 0.41 | 1.74 | 0.00 | Sig |
| **29** | 1.03 | 0.35 | 1.71 | 0.00 | Sig |

The results showed that after excluding any of the studies in the RPE, the overall effect value changed only slightly and was statistically significant (p＜0.05).
