# Supplementary material for: The effect of re-warm up and its moderators on performance enhancement and rating of perceived exertion in team-sport athletes: A systematic review and three-level meta-analysis
Source: PLoS One. 2026 Sep 18;21(9):e0358479. doi: 10.1371/journal.pone.0358479 (PMC13588333; doi:10.1371/journal.pone.0358479)
Supplement: Supplementary file 8 [file pone.0358479.s008.docx]

**S8 File.** **Results of traditional two-level meta-analyses and corresponding forest plots.**

**Figure 1 Forest plot of the effect of RWU on CMJ**

**Figure 2 Forest plot of the effect of RWU on SJ**

**Figure 3 Forest plot of the effect of RWU on Sprint time**

**Figure 4 Forest plot of the effect of RWU on RSA**

**Figure 5 Forest plot of the effect of RWU on agility**

**Figure 6 Forest plot of the effect of RWU on max strength**

**Figure 7 Forest plot of the effect of RWU on RPE**
