# Supplementary material for: The effect of re-warm up and its moderators on performance enhancement and rating of perceived exertion in team-sport athletes: A systematic review and three-level meta-analysis
Source: PLoS One. 2026 Sep 18;21(9):e0358479. doi: 10.1371/journal.pone.0358479 (PMC13588333; doi:10.1371/journal.pone.0358479)
Supplement: Supplementary file 9 [file pone.0358479.s009.docx]

**S9 File. Moderator analyses, including continuous-variable analyses, RWU exercise-type analyses, and multivariable moderator analyses.**

**9.1 Continuous variable analysis**

| **Indicators** | **Variables** | **K (N)** | **β** | **t_1_** | **σ²-2** | **σ²-3** | **P_m_** |
| --- | --- | --- | --- | --- | --- | --- | --- |
| **CMJ** | BMI | 11（15） | -0.12 | -0.74 | 0.36 | 0.42 | 0.47 |
|  | Intervention time | 11（15） | 0.01 | 0.02 | 0.38 | 0.43 | 0.98 |
| **SJ** | BMI | 4（13） | -0.12 | -0.14 | 0.26 | 0.43 | 0.90 |
|  | Intertime | 4（13） | 0.63 | 2.75 | 0.24 | 0.00 | 0.02 |
|  | Training age | 3（12） | -0.21 | -0.35 | 0.28 | 0.66 | 0.73 |
| **Speed** | BMI（spt） | 8（17） | 0.11 | 1.06 | 0.00 | 0.20 | 0.31 |
|  | BMI（RSA） | 3（8） | 0.10 | 1.20 | 0.00 | 0.00 | 0.28 |
|  | Intervention time（spt） | 8（17） | -0.18 | -1.27 | 0.00 | 0.25 | 0.22 |
|  | intervention time（RSA） | 3（8） | -0.12 | -0.77 | 0.00 | 0.15 | 0.47 |
| **Agility** | BMI | 6(12) | -0.19 | -1.25 | 0.00 | 0.05 | 0.24 |
|  | Intervention time | 6(12) | -0.20 | -1.11 | 0.00 | 0.06 | 0.29 |
|  | Training age | 6(12) | 0.01 | 0.17 | 0.00 | 0.09 | 0.87 |
| **RPE** | BMI | 12（29） | -0.33 | -1.98 | 0.15 | 0.82 | 0.06 |
|  | Intertime | 12（29） | -0.15 | -1.04 | 0.14 | 1.13 | 0.31 |

**Annotation：**Intertime: intervention time

**9.2 RWU exercise types variable analysis**

| **Indicators** | **Variables** | **g** | **P_g_** | **CI** | **σ²-2** | **σ²-3** | **P_m_** |
| --- | --- | --- | --- | --- | --- | --- | --- |
| **Sprint time** | tyitype | -0.928 | 0.001 | -1.425,-0.430 | 0.000 | 0.216 | 0.395 |
|  | notyitype | -0.603 | 0.075 | -1.275, 0.068 | 0.000 | 0.216 |  |
| **SJ** | tyitype | 0.845 | 0.075 | -0.100, 1.791 | 0.263 | 0.328 | 0.525 |
|  | notyitype | 0.285 | 0.707 | -1.339, 1.909 | 0.263 | 0.328 |  |
| **CMJ** | tyitype | 1.091 | 0.006 | 0.352, 1.831 | 0.384 | 0.331 | 0.322 |
|  | notyitype | 0.650 | 0.042 | 0.025, 1.276 | 0.384 | 0.331 |  |
| **Agility** | tyitype | -0.513 | 0.014 | -0.895, -0.131 | 0.000 | 0.066 | 0.708 |
|  | notyitype | -0.396 | 0.181 | -1.009, 0.218 | 0.000 | 0.066 |  |

**Annotation：** “tyitype" refers to the use of specific warm-up activities related to sports performance; “notyitype” refers to the use of general warm-up activities that are not related to sports performance; **P_g_:** refers to statistical significance compared to the control group; P_m_: refers to the statistical significance of the moderating effect.

**9.3 Multivariable Analysis**

**Table 1 Multivariate moderating effects**

| **Indicators** | **multiplemoderator** | **Variables** | ***g/β*** | **95% CI** | **P_g_** | **σ²-2** | **σ²-3** | **P_m_** |
| --- | --- | --- | --- | --- | --- | --- | --- | --- |
| **Sprint time** | Running distance Interval format | without interval+30m | -0.34 | -1.16, 0.48 | 0.39 | 0.00 | 0.116 | 0.21 |
|  |  | without interval+5-10m | -0.44 | -1.06,0.17 | 0.15 | 0.00 | 0.116 | 0.21 |
|  |  | without interval+20m | -0.40 | -1.01,0.21 | 0.19 | 0.00 | 0.116 | 0.21 |
|  |  | with interval +30m | -1.06 | -1.92,-0.20 | 0.02 | 0.00 | 0.116 | 0.21 |
|  |  | with interval +20m | -1.11 | -1.68,-0.56 | <0.001 | 0.00 | 0.116 | 0.21 |
|  |  | with interval +5-10m | -1.16 | -1.75,-0.57 | <0.001 | 0.00 | 0.116 | 0.21 |
|  | running distance+ Interval format+age group | without interval+20m+adult | 0.07 | -0.52,0.66 | 0.80 | 0.00 | 0.00 | 0.01 |
|  |  | with interval + 20 m + adult | -0.82 | -1.29,-0.36 | 0.002 | 0.00 | 0.00 | 0.01 |
|  |  | with interval + 30m+ adult | -0.93 | -1.68,-0.18 | 0.02 | 0.00 | 0.00 | 0.01 |
|  |  | without interval + 30m+ adult | -0.03 | -0.79,0.72 | 0.93 | 0.00 | 0.00 | 0.01 |
|  |  | without interval+5-10m+ adult | -0.14 | -0.60,0.32 | 0.51 | 0.00 | 0.00 | 0.01 |
|  |  | with interval+5-10m+ adult | -1.04 | -1.46,-0.61 | <.001 | 0.00 | 0.00 | 0.01 |
|  |  | interval+5-10m+ adolescent | -1.57 | -2.20, -0.94 | <.001 | 0.00 | 0.00 | 0.01 |
|  |  | without interval+5-10m+adolescent | -0.68 | -1.18,-0.17 | 0.01 | 0.00 | 0.00 | 0.01 |
|  |  | without interval + 30m+ adolescent | -0.57 | -1.17, 0.03 | 0.06 | 0.00 | 0.00 | 0.01 |
|  |  | with interval + 30m+ adolescent | -1.46 | -2.18,-0.74 | <.001 | 0.00 | 0.00 | 0.01 |
|  |  | with interval + 20m+ adolescent | -1.36 | -1.75, -0.97 | <.001 | 0.00 | 0.00 | 0.01 |
|  |  | without interval + 20m+ adolescent | -0.46 | -0.80, -0.13 | 0.01 | 0.00 | 0.00 | 0.01 |
| **CMJ** | takeoff style + lower limb dominance | CMJnd+unilateral | 1.94 | 1.00, 2.87 | <.001 | 0.295 | 0.068 | 0.002 |
|  |  | CMJd+unilateral | 2.52 | 1.55, 3.48 | <.001 | 0.295 | 0.068 | 0.002 |
|  | takeoff style + lower limb dominance+ Interval format | bilateral+ without interval | 1.06 | 0.42, 1.70 | 0.003 | 0.338 | 0.00 | < .001 |
|  |  | unilateral + without interval | 2.46 | 1.85, 3.08 | <.001 | 0.338 | 0.00 | < .001 |
|  |  | CMJd+ unilateral + without interval | 2.77 | 1.88, 3.67 | <.001 | 0.345 | 0.00 | < .001 |
|  |  | CMJnd+ unilateral + without interval | 2.18 | 1.32, 3.04 | <.001 | 0.345 | 0.00 | < .001 |
|  |  | bilateral+ with interval | 0.55 | 0.16, 0.94 | 0.01 | 0.320 | 0.00 | < .001 |
|  |  | unilateral+ with interval | 1.95 | 1.00, 2.91 | <.001 | 0.320 | 0.00 | < .001 |
|  |  | CMJnd+unilateral+ with interval | 1.67 | 0.55, 2.80 | <.001 | 0.326 | 0.00 | < .001 |
|  |  | CMJd+unilateral+ with interval | 2.26 | 1.11, 3.41 | <.001 | 0.326 | 0.00 | < .001 |
|  | takeoff style + Interval format + age group | unilateral+with interval+ adolescent | 2.01 | 0.98, 3.04 | <.001 | 0.343 | 0.006 | < .001 |
|  |  | unilateral+without interval+adolescent | 2.40 | 1.67, 3.12 | <.001 | 0.33 | 0.038 | 0.004 |
|  |  | bilateral+without interval +adolescent | 1.11 | 0.39, 1.82 | 0.004 | 0.33 | 0.038 | 0.004 |
|  |  | bilateral+ with interval +adolescent | 0.64 | 0.05, 1.23 | 0.04 | 0.343 | 0.006 | < .001 |
|  |  | bilateral+with interval+ adult | 0.49 | -0.03, 1.01 | 0.06 | 0.343 | 0.006 | < .001 |
|  |  | unilateral+ with interval+ adult | 1.86 | 0.78, 2.94 | 0.002 | 0.343 | 0.006 | < .001 |
|  |  | unilateral+ without interval + adult | 1.96 | 0.79, 3.12 | 0.002 | 0.33 | 0.038 | 0.004 |
|  |  | bilateral+ without interval + adult | 0.67 | -0.36, 1.70 | 0.19 | 0.33 | 0.038 | 0.004 |
|  | Interval format + intensity | without interval+Mintensity | 1.561 | 0.463, 2.660 | 0.008 | 0.442 | 0.320 | 0.595 |
|  |  | with interval+Mintensity | 0.764 | 0.021, 1.507 | 0.045 | 0.441 | 0.259 | 0.429 |
|  |  | without interval+MHintensity | 0.994 | -1.072, 3.060 | 0.327 | 0.442 | 0.320 | 0.595 |
|  |  | with interval+MHintensity | 0.372 | -1.215, 1.958 | 0.631 | 0.441 | 0.259 | 0.429 |
|  |  | without interval+Hintensity | 1.129 | -0.017, 2.276 | 0.053 | 0.442 | 0.320 | 0.595 |
|  |  | with interval+Hintensity | 0.442 | -0.489, 1.372 | 0.335 | 0.441 | 0.259 | 0.429 |
| **Agility** | Intertime+ age group + Interval format | Intertime（fixed） | -0.35 | -0.70, -0.00 | 0.05 | 0.000 | 0.000 | 0.1 |
|  | age group + Interval format | without interval + adolescent | -0.61 | -1.21, -0.02 | 0.05 | 0.000 | 0.080 | 0.6 |
|  |  | without interval + adult | -0.92 | -2.03, 0.19 | 0.09 | 0.000 | 0.080 | 0.6 |
|  |  | with interval+ adult | -0.58 | -1.29, 0.13 | 0.10 | 0.000 | 0.080 | 0.6 |
|  |  | with interval+ adolescent | -0.27 | -0.88, 0.35 | 0.35 | 0.000 | 0.080 | 0.6 |
| **RPE** | age group + Effect category | Non-immediate+adult | 0.30 | -0.30, 0.89 | 0.31 | 0.040 | 0.484 | < .001 |
|  |  | Immediate+adult | 0.91 | 0.39, 1.44 | 0.001 | 0.040 | 0.484 | < .001 |
|  |  | Non-immediate+ adolescent | 2.23 | 0.94, 3.52 | 0.001 | 0.040 | 0.484 | < .001 |
|  |  | Immediate + adolescent | 2.85 | 1.65, 4.04 | <.001 | 0.040 | 0.484 | < .001 |
|  | age group + Effect category + Interval format | Non-immediate+with interval+adolescent | 2.20 | 0.88, 3.51 | 0.002 | 0.039 | 0.515 | 0.003 |
|  |  | Non-immediate +without interval+adolescent | 2.61 | 0.92, 4.31 | 0.004 | 0.039 | 0.515 | 0.003 |
|  |  | Immediate+without interval+adolescent | 3.26 | 1.59, 4.93 | <.001 | 0.039 | 0.515 | 0.003 |
|  |  | Immediate + with interval+adolescent | 2.84 | 1.62, 4.07 | <.001 | 0.039 | 0.515 | 0.003 |
|  |  | Immediate+with interval+ adult | 0.80 | 0.17, 1.43 | 0.02 | 0.039 | 0.515 | 0.003 |
|  |  | Immediate +without interval+ adult | 1.22 | 0.24, 2.20 | 0.02 | 0.039 | 0.515 | 0.003 |
|  |  | Non-immediate +without interval+ adult | 0.57 | -0.39, 1.54 | 0.23 | 0.039 | 0.515 | 0.003 |
|  |  | Non-immediate +with interval+ adult | 0.15 | -0.56, 0.87 | 0.67 | 0.039 | 0.515 | 0.003 |
|  | BMI + Effect category + Interval format + intensity | BMI（Effect category + with interval format+Lintensity） | -0.34 | -0.65, -0.04 | 0.03 | 0.027 | 0.693 | 0.011 |
|  |  | BMI（Effect category + with interval format+Mintensity） | -0.37 | -0.70, -0.03 | 0.03 | 0.040 | 0.764 | 0.021 |
|  |  | BMI（Effect category + with interval format+Hintensity） | -0.26 | -0.59, 0.07 | 0.11 | 0.025 | 0.666 | 0.009 |

**Annotation：CMJd:** CMJ with dominant leg; **CMJnd:** CMJ with non-dominant leg; **Non-immediate:** The effect of the 5~45min period in the second half; **Immediate:** Immediate effect after RWU; **Mintensity:** moderate intensity; **MHintensity:** moderate to high intensity; **Hintensity**: high intensity; **P_g_:** refers to statistical significance compared to the control group; P_m_: refers to the statistical significance of the moderating effect.
