# Supplementary material for: The effect of re-warm up and its moderators on performance enhancement and rating of perceived exertion in team-sport athletes: A systematic review and three-level meta-analysis
Source: PLoS One. 2026 Sep 18;21(9):e0358479. doi: 10.1371/journal.pone.0358479 (PMC13588333; doi:10.1371/journal.pone.0358479)
Supplement: Supplementary file 10 [file pone.0358479.s010.docx]

**S10 File.Sensitivity analyses using different assumed correlation coefficients.**

| **Outcome indicators** | **R** | **estimate** | **95% CI** | **p** |
| --- | --- | --- | --- | --- |
| acceleration and speed | 0.3 | -0.69 | [-1.038,-0.342] | <.001 |
|  | 0.5 | -0.817 | [-1.231,-0.403] | <.001 |
|  | 0.7 | -1.045 | [-1.586,-0.504] | <.001 |
| Agility | 0.3 | -0.432 | [-0.747,-0.117] | 0.012 |
|  | 0.5 | -0.484 | [-0.82,-0.149] | 0.009 |
|  | 0.7 | -0.577 | [-0.945,-0.209] | 0.005 |
| jumps | 0.3 | 0.612 | [0.162,1.063] | 0.01 |
|  | 0.5 | 0.819 | [0.294,1.345] | 0.004 |
|  | 0.7 | 0.844 | [0.295,1.394] | 0.004 |
| Jump - sj | 0.3 | 0.729 | [-0.018,1.475] | 0.055 |
|  | 0.5 | 0.807 | [0.019,1.594] | 0.045 |
|  | 0.7 | 0.94 | [0.106,1.775] | 0.031 |
| RPE | 0.3 | 0.913 | [0.321,1.505] | 0.004 |
|  | 0.5 | 1.046 | [0.382,1.71] | 0.003 |
|  | 0.7 | 1.243 | [0.467,2.019] | 0.003 |
| RSA | 0.3 | 0.214 | [-0.347,0.774] | 0.398 |
|  | 0.5 | -0.345 | [-0.749,0.06] | 0.084 |
|  | 0.7 | 0.395 | [-0.358,1.148] | 0.255 |
| strengths | 0.3 | 0.172 | [-0.873,1.217] | 0.552 |
|  | 0.5 | 0.203 | [-0.844,1.249] | 0.493 |
|  | 0.7 | 0.172 | [-0.873,1.217] | 0.552 |
