# Supplementary material for: The effect of re-warm up and its moderators on performance enhancement and rating of perceived exertion in team-sport athletes: A systematic review and three-level meta-analysis
Source: PLoS One. 2026 Sep 18;21(9):e0358479. doi: 10.1371/journal.pone.0358479 (PMC13588333; doi:10.1371/journal.pone.0358479)
Supplement: Supplementary file 11 [file pone.0358479.s011.docx]

**S11 File. Detailed re-warm up (RWU) protocol extraction table and extraction-consensus records .**

| **Study ID** | **RWU Type** | **Duration** | **Strength** | **Intermittent format** | **Organizational Model** | **Extractor1** | **Extractor2** | **consistent** | **Reasons for the Disagreement** | **Third-Party Arbitration Ruling** |
| --- | --- | --- | --- | --- | --- | --- | --- | --- | --- | --- |
| Christaras 2023 | Specificity | 3 min | High (70–90% of maximum speed) | Non-stop | 12minBreak+3min RWU | J-Z | HL-T | Yes | —— | —— |
| Edholm 2014 | Non-specific | 7 min | Low to moderate (~70% of maximum heart rate) | Intermittent | 7minBreak+7min RW+1minBreak | J-Z | HL-T | Yes | —— | —— |
| Koutsouridis 2024 | Non-specific | 3 min | Moderate-to-low (40% VO₂max) | Non-stop | 11minBreak+3min RW | J-Z | HL-T | Yes | —— | —— |
| Fashioni 2020 | Specificity | 3 min | High | Non-stop | 12minBreak+3min RW | J-Z | HL-T | Yes | —— | —— |
| Ramos-Campo 2020 | Specificity | 4.5 min | Upper-middle | Intermittent | 7.5minBreak+4.5min RW+3minBreak | J-Z | HL-T | No | Is the horizontal jump considered a sport-specific exercise for football? | Included; the RWU type is "specific." While the transferability of enhanced training for football players is questionable, this is how it is classified in the original text. |
| Lovell 2011 | Specificity | 5 min | Moderate | Intermittent | 9minBreak+5min RW+1minBreak | J-Z | HL-T | No | Does whole-body vibration count as RWU? | Included; RWU type: "Specific"; whole-body vibration as a device-assisted warm-up method—although different from traditional dynamic warm-ups—was included in the original study and produced an effect size. |
| Ltifi 2023 | Specificity | 3 min | High | Intermittent | 10minBreak+3min RW+2minBreak | J-Z | HL-T | Yes | —— | —— |
| Matsentides 2023 | Specificity | 4 min | High | Intermittent | 8minBreak+4min RW+3minBreak | J-Z | HL-T | Yes | —— | —— |
| Mohr 2004 | Non-specific | 7 min | Moderate | Intermittent | 7minBreak+7min RW+1minBreak | J-Z | HL-T | Yes | —— | —— |
| Sanchez-Sanchez 2024 | Specificity | 5 min | Low to medium | Non-stop | 10minBreak+5min RW | J-Z | HL-T | Yes | —— | —— |
| Tong 2019 | Non-specific | 4 min | Low to medium | Non-stop | 11minBreak+4min RW | J-Z | HL-T | No | Does core training with inhalation resistance count as RWU? | Included; the RWU type is "non-specific." Although there was no hyperthermic effect, the original text notes "re-warm up," and the study was included in the analysis. |
| Yamashita 2022 | Non-specific | 2 min | High | Intermittent | 12minBreak+2min RW+1minBreak | J-Z | HL-T | No | Is cycling considered a team sport? | Included; the sport category is "set in a team sport"; tested separately in the subgroup analysis |
| Yanaoka 2018a | Non-specific | 7 min/3 min | Medium (70% of maximum heart rate) | Intermittent | 7min/11minBreak+7min/3min RW+1minBreak | J-Z | HL-T | No | How do I extract the two durations? | Extract separately, using 7 minutes and 3 minutes as two independent conditions, and calculate the effect sizes for each. |
| Yanaoka 2018b | Non-specific | 3 min | Moderate (30% or 60% VO₂max) | Intermittent | 11minBreak+3min RW+1minBreak | J-Z | HL-T | No | How are the two types of strength classified? | 30% and 60% of VO₂max were selected as two independent conditions |
| Yanaoka 2021 | Specificity | 1 min | High (90% VO₂max) | Intermittent | 14minBreak+1min RW | J-Z | HL-T | Yes | —— | —— |
| Yanaoka 2020 | Non-specific | 3 min/1 min | Low/High (30% or 90% VO₂max) | Intermittent | 11min/13minBreak+3min/1min RW+1minBreak | J-Z | HL-T | No | How do I extract both conditions? | Extract separately, using 3 minutes of low intensity and 1 minute of high intensity as two independent conditions |
| González-Devesa 2023 | Specificity | 3 min | High | Intermittent | 6minBreak+3min RW+1minBreak | J-Z | HL-T | Yes | —— | —— |
| Flórez-Gil 2025 | Specificity | 1 min | Low to medium | Non-stop | 2minBreak+1min RW Loop | J-Z | HL-T | Yes | —— | —— |
