# Supplementary material for: The effect of re-warm up and its moderators on performance enhancement and rating of perceived exertion in team-sport athletes: A systematic review and three-level meta-analysis
Source: PLoS One. 2026 Sep 18;21(9):e0358479. doi: 10.1371/journal.pone.0358479 (PMC13588333; doi:10.1371/journal.pone.0358479)
Supplement: Supplementary file 12 [file pone.0358479.s012.docx]

**S12 File.** **Subgroup analyses and comparisons across different sports**

| **Outcome indicators** | **Sports** | **Hedges' g** | **95%CI** | **p** |
| --- | --- | --- | --- | --- |
| acceleration and speed | Rugby | -0.585 | [-1.124, 1.686] | 0.676 |
| jump | Basketball | 0.73 | [-1.423, 0.982] | 0.707 |
|  | Rugby | 0.372 | [-2.516, 1.357] | 0.541 |
| Jump - sj | Basketball | 0.468 | [-2.598, 1.812] | 0.7 |
| Agility | Basketball | -0.456 | [-0.696, 0.782] | 0.899 |
| RPE | Basketball | 0.844 | [-3.257, 1.308] | 0.386 |
|  | Rugby | 0.158 | [-4.851, 1.531] | 0.293 |
|  | Cue ball | 0.517 | [-4.502, 1.901] | 0.409 |
|  | Bicycle | 1.099 | [-3.226, 1.787] | 0.558 |
|  | Mixed Team | 0.806 | [-3.524, 1.501] | 0.413 |
| RSA | Cue ball | -0.143 | [-0.669, 1.525] | 0.362 |
|  | Rugby | -0.586 | [-1.295, 1.265] | 0.977 |
| strength | Basketball | 0.173 | [-6.916, 6.716] | 0.883 |
