# Supplementary material for: The effect of re-warm up and its moderators on performance enhancement and rating of perceived exertion in team-sport athletes: A systematic review and three-level meta-analysis
Source: PLoS One. 2026 Sep 18;21(9):e0358479. doi: 10.1371/journal.pone.0358479 (PMC13588333; doi:10.1371/journal.pone.0358479)
Supplement: Supplementary file 13 [file pone.0358479.s013.docx]

**S13 File.** **Comparative analyses of simulated and real competitive environments.**

| **Outcome indicators** | **Hedges' g** | **95%CI** | **p** |
| --- | --- | --- | --- |
| acceleration and speed | -0.792 | [-1.016, 1.127] | 0.913 |
| jumps | 0.995 | [-1.112, 1.565] | 0.73 |
| Agility | 0.295 | [-2.185, 0.195] | 0.092 |
| junmps | -0.241 | [-0.433, 1.092] | 0.358 |
| RPE | 1.024 | [-1.649, 1.589] | 0.97 |
| RSA | -0.29 | [-0.782, 1.344] | 0.542 |
